# The Cell and the Sum of Its Parts: Patterns of Complexity in Biosignatures as Revealed by Deep UV Raman Spectroscopy

**DOI:** 10.3389/fmicb.2019.00679

**Published:** 2019-05-14

**Authors:** Haley M. Sapers, Joseph Razzell Hollis, Rohit Bhartia, Luther W. Beegle, Victoria J. Orphan, Jan P. Amend

**Affiliations:** ^1^Division of Geological and Planetary Sciences, California Institute of Technology, Pasadena, CA, United States; ^2^Jet Propulsion Laboratory, California Institute of Technology, Pasadena, CA, United States; ^3^Department of Earth Sciences, University of Southern California, Los Angeles, CA, United States; ^4^Department of Biological Sciences, University of Southern California, Los Angeles, CA, United States

**Keywords:** biosignatures, deep UV Raman spectroscopy, Mars 2020, SHERLOC, spectral deconvolution

## Abstract

The next NASA-led Mars mission (Mars 2020) will carry a suite of instrumentation dedicated to investigating Martian history and the *in situ* detection of potential biosignatures. SHERLOC, a deep UV Raman/Fluorescence spectrometer has the ability to detect and map the distribution of many organic compounds, including the aromatic molecules that are fundamental building blocks of life on Earth, at concentrations down to 1 ppm. The mere presence of organic compounds is not a biosignature: there is widespread distribution of reduced organic molecules in the Solar System. Life utilizes a select few of these molecules creating conspicuous enrichments of specific molecules that deviate from the distribution expected from purely abiotic processes. The detection of far from equilibrium concentrations of a specific subset of organic molecules, such as those uniquely enriched by biological processes, would comprise a universal biosignature independent of specific terrestrial biochemistry. The detectability and suitability of a small subset of organic molecules to adequately describe a living system is explored using the bacterium *Escherichia coli* as a model organism. The DUV Raman spectra of *E. coli* cells are dominated by the vibrational modes of the nucleobases adenine, guanine, cytosine, and thymine, and the aromatic amino acids tyrosine, tryptophan, and phenylalanine. We demonstrate that not only does the deep ultraviolet (DUV) Raman spectrum of *E. coli* reflect a distinct concentration of specific organic molecules, but that a sufficient molecular complexity is required to deconvolute the cellular spectrum. Furthermore, a linear combination of the DUV resonant compounds is insufficient to fully describe the cellular spectrum. The residual in the cellular spectrum indicates that DUV Raman spectroscopy enables differentiating between the presence of biomolecules and the complex uniquely biological organization and arrangements of these molecules in living systems. This study demonstrates the ability of DUV Raman spectroscopy to interrogate a complex biological system represented in a living cell, and differentiate between organic detection and a series of Raman features that derive from the molecular complexity inherent to life constituting a biosignature.

## Introduction

The search for life beyond Earth has motivated decades of planetary exploration from the first life detection experiments on board the Viking missions to Mars in 1975 (Klein et al., 1972; Soffen and Snyder, 1976; Levin and Straat, 1977) to current exploration by the Mars Science Laboratory (Summons et al., 2011; Grotzinger et al., 2012) to future missions to potentially habitable icy bodies in our Solar System (e.g., Phillips and Pappalardo, 2014). Mars is the closest, potentially habitable body in the Solar System (e.g., Farmer and Des Marais, 1999; Des Marais, 2010; Grotzinger et al., 2013; Filiberto et al., 2014), and near-term missions including Mars 2020 (Mustard et al., 2013; Williford et al., 2018) and ExoMars (Vago et al., 2017) seek to understand the distribution of habitability criteria, such as the spatial and temporal extent of liquid water and availability of carbon and energy sources, in addition to the detection and evaluation of putative biosignatures. Mars 2020 will carry a suite of instrumentation capable of detecting and characterizing geochemical and organic patterns in a variety of samples that will be collected from pre-characterized sites using available orbital datasets and cached for eventual sample return and further evaluation for evidence of life (Williford et al., 2018).

On the Mars 2020 payload, the Scanning Habitable Environments with Raman and Luminescence for Organic and Chemicals (SHERLOC); a list of abbreviations and acronyms is provided below, is a DUV Raman spectrometer capable of mapping organic compositions of target samples (Beegle et al., 2015). Relevant to this study, SHERLOC has the ability to detect the Raman peaks of organic molecules *in situ* at concentrations below 0.1 wt% in a 100 micron area and 10-5 ww over an observed area of 5 mm × 5 mm (Abbey et al., 2017). This includes the nucleobases and amino acids known to be essential components of terrestrial life. Furthermore, these concentrations can be mapped across a surface in order to show spatial distributions. However, the mere presence of organic compounds is not evidence of life – here we demonstrate a spectral threshold for biogenicity based on the Raman active components within a cell compared to the Raman spectra of the cell itself. These results provide a context for the interpretation of DUV Raman spectra of organic molecules collected by SHERLOC on Mars 2020 as potential biosignatures.

The formation of a biosignature requires that a biological process results in the accumulation of a biogenic ‘signal’ that differs significantly from the background abiotic ‘noise.’ Detection requires that the signal is in high enough concentration, or chemically and physically distinct enough from the background environment that it is both distinguishable and not subject to attrition (Des Marais et al., 2008; Des Marais, 2013; Hays et al., 2017). Raman spectroscopy can be used to detect the unique biosignature of a cell as the enrichment of specific organic molecules, in the same location with sufficient structural complexity that cannot be adequately explained by known abiotic processes. In this paper we are focused on the analysis of a single DUV Raman spectrum obtained from *Escherichia coli* cells harvested during exponential growth, without further spatial or mineralogical context, to determine if it is possible to distinguish the unique chemical biosignature of those cells from their DUV resonant molecular components alone. While minor variations in Raman spectra have been used to differentiate different microbial species (Huang et al., 2004; Pahlow et al., 2015), the dominant vibrational modes are shared reflecting similar macromolecular compositions in other bacterial cells (Wu et al., 2001), viruses (Wen and Thomas, 1998), and eukaryotic cells (Kumamoto et al., 2012).

While the chemical structure of abiotically synthesized and biogenically produced organic molecules do not differ, the distribution and co-occurrence patterns of the particular compounds is significant. Life exploits boundary conditions to harness energy and as such its distribution reflects this. Life is not homogenous: complexity in distribution is a fundamental property of life (Bhartia et al., 2010). Distribution can be described by two parameters: spatial and constituent. The inventory of organic molecules is significant as it reflects the selectivity of uniquely biological processes. The spatial distribution of these molecules is also significant: the presence of nucleobases and proteinaceous amino acids within the same sample is not necessarily a biosignature, however, a conspicuous enrichment co-occurring spatially and temporally is difficulty to reconcile abiotically. These reduced organic carbon molecules are the fundamental building blocks of terrestrial life; however, they are not unique to life. Nucleobases including adenine, guanine, and uracil have been found in the Murchison, Murray and Orgueil Martian meteorites (Stoks and Schwartz, 1979; Martins et al., 2008; Steele et al., 2016) and over 80 amino acids, including 55 α-amino acids, have been detected in carbonaceous chondrites (Sephton, 2002; Sephton and Botta, 2005; Pizzarello et al., 2006; Schmitt-Kopplin et al., 2010; Burton et al., 2012). Of nearly 4000 plausible α-amino acids structures (Meringer et al., 2013), only ∼700 have been isolated from biological systems (Hunt, 1985). From these 700 amino acids, only 20 are translationally encoded in all lineages of terrestrial life (Wong, 1975; Hardy, 1985). Only 8 of the 20 amino acids comprising the standard genetic code have been observed in extraterrestrial samples (Pizzarello et al., 2006; Burton et al., 2012) and only about half of the translationally encoded amino acids can be accounted for experimentally via abiotic synthesis and prebiotic simulations (Miller, 1953; Muñoz Caro et al., 2002; Johnson et al., 2008; Higgs and Pudritz, 2009; Cleaves, 2010; Parker et al., 2011). These observations have divided the 20 universally translated amino acids into early and late groups (Wong, 1975, 2005; Higgs and Pudritz, 2009). The former represents simple amino acids that can be formed prebiotically through abiotic processes comprising the earliest genetic code. The latter group were incorporated into the genetic code following the evolution of biosynthetic pathways modifying simpler precursors (Wong, 1975). Calculations of the Gibbs free energy of formation for each of the 20 proteinaceous amino acids indicate that the latter group requires a significantly higher energy cost (Amend and Shock, 1998) and to date these amino acids have not been observed in extraterrestrial materials implying their presence requires biosynthetic pathways. Thus an organic biosignature is not simply the enrichment of a specific subset of organic molecules, but that the molecules enriched display a structural complexity not explained or expected to be produced by purely abiotic processes. As recognized by Nelson et al. (1992), ‘the cell is more than the sum of its parts’; we leverage this to illustrate a spectral distinction between a collection of organic molecules and those that comprise a living system.

**Figure 1:**
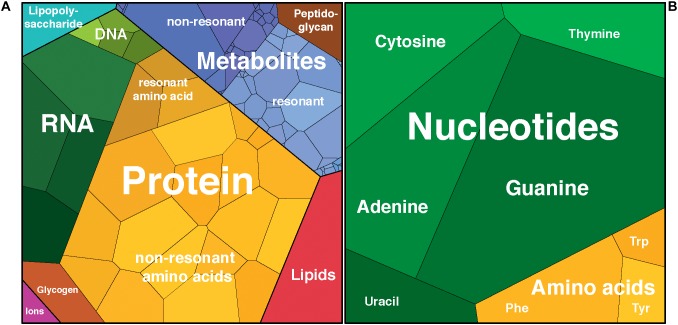
Schematic representation of **(A)** cell components by dry mass and **(B)** integrated Raman intensities from deconvolution of the *Escherichia coli* Raman spectrum using nucleotide and amino acid spectra. Proportional visualization using Voronoi diagrams with the area of each cell representing the relative contribution of that component to the total. Plots rendered using Proteomaps http://bionic-vis.biologie.uni-greifswald.de/ (Bernhardt et al., 2009; Otto et al., 2010; Liebermeister et al., 2014).

Identifying complex mixtures of similar compounds *in situ* is challenging, as many analytical techniques either consume the sample and employ chromatographic separation methods or can only probe bulk composition at length scales far beyond that of individual cells. Assessing the biogenicity of a putative biosignature requires multiple, complementary analytical techniques and contextual information including spatial distribution, destructive and bulk methods are limited. Raman spectroscopy offers the necessary sensitivity to chemical structure, at spatial resolutions comparable to the size of a cell, without destroying the sample. By using DUV excitation, we can also exploit the combined signal enhancement of both high-frequency excitation and molecular resonance with opto-electronic transitions (Nelson et al., 1992; and references therein; Tarcea et al., 2007). This enables the identification of aromatic components within cellular materials even at very low concentrations that would otherwise be undetectable using more conventional excitation wavelengths, such as the 532 and 633 nm lasers employed in Green and Red Raman respectively (Beegle et al., 2015; and references therein). The Raman scattering intensity is related to excitation frequency such that high frequency excitation leads to a greater proportion of Raman-scattered light for a given laser power (Long, 1977). Using DUV excitation also provides resonance with the π-π^∗^ absorption band of many aromatic molecules, including the nucleic acids and some amino acids, leading to an overall increase in scattering cross-section of up to 10,000x (Asher and Johnson, 1984; Asher and Murtaugh, 1988; Ianoul et al., 2002) vs. non-resonant, lower-frequency excitation. Resonance provides particular sensitivity to minor conformational and structural changes that involve the aromatic ring (Asher, 1993; Toyama et al., 1999), and resonant Raman has been used previously to probe molecular conformers, intermolecular packing, and photo-oxidation reactions in aromatic compounds (Razzell-Hollis et al., 2014; Wade et al., 2017; Wood et al., 2017). Identification of molecular structures by the pattern of peaks in the Raman spectrum is made more challenging when several similar molecules are present together, as the identifying peaks of one molecule may overlap with modes from others. However, by using DUV excitation to resonantly enhance signals from aromatic molecules, we can reduce the number of detectable molecules to a smaller subset that still constitute a distinctive biosignature. For terrestrial cells this subset has been established to consist of the five nucleobases and three aromatic amino acids (AAAs) (Britton et al., 1988; Nelson et al., 1992; Chadha et al., 1993). We therefore define a set of molecular standards based on these eight aromatic molecules (Figure 1).

By using *E. coli* as a model organism, we can demonstrate that not only does its DUV Raman spectrum reflect the enrichment of specific aromatic molecules, but that molecular complexity, i.e., spectra from nucleotides rather than simple nucleobases, is required to deconvolute the cellular spectrum. We also illustrate the ability of DUV Raman spectroscopy to differentiate between the spectrum of a cell and a representative artificial mixture of its Raman resonant components, i.e., whether the cell is more than the sum of its parts and if this itself constitutes a distinctive biosignature. Here we present an illustration of the importance of structural complexity in biosignatures by systematically increasing the complexity of biomolecules to deconvolute the DUV Raman spectra of *E. coli* into its constituent DUV resonant components.

## Materials and Methods

### *Escherichia coli* Cultures

*Escherichia coli* K12 was grown from frozen stocks overnight in 1 mL of defined minimal media (M9) containing 0.4% glucose, 47.6 mM Na_2_HPO_4_, 22.06 mM KH_2_PO_4_, 8.56 mM NaCl, 18.7 mM NH_4_Cl, 99.12 μM CaCl_2_ and 0.1 mg/L thiamine, pH adjusted to 7.0 and filtered sterilized through a 0.22 μm PES membrane filter. Cultures were incubated in a shaker at 37°C and were propagated to a sufficient volume for subsequent sampling. Cells were further transferred three times during mid-log growth as determined by measuring the absorbance at 600 nm (OD_600_) using a DR-2700 spectrophotometer (Hach, Inc.). Triplicate 150 mL cultures were established and cells were harvested aseptically after 4 h during exponential growth and fixed with 4% paraformaldehyde for 1 h at room temperature. It has been noted that fixation does not influence the cellular spectra and also prevents spectral changes due to radiation-induced stress observed in live cells (Kumamoto et al., 2011). Following fixation cells were pelleted, washed twice in phosphate buffered saline (PBS), resuspended in 50% PBS, and finally re-suspended in MilliQ H_2_O to an OD_600_ of 0.2 (1.6 × 10^8^ cells/ml) based on the initial optical density reading. 2 μL of the washed and re-suspended sample was spotted onto a sterile aluminum wafer (Multipurpose 6061, McMaster-Carr) and allowed to air dry prior to Raman analysis. Given a laser diameter of approximately 68 μm and a dry spot with a diameter of 2 mm, each laser spot would interrogate ∼370 cells, assuming a roughly equal distribution of cells. A 50 μl droplet of cell suspension was also measured with Raman immediately to assess spectral artifacts produced by drying. The DUV Raman spectrum of the aluminum wafer displayed no intrinsic vibrational modes (Supplementary Figure S1).

### Molecular Standards

Samples of the nucleobases adenine (Sigma, A8751), cytosine (Sigma, C3506), guanine (Aldrich, G11950), thymine (Sigma, T0376), uracil (Alfa Aesar, A15570), and the amino acids phenylalanine (Sigma, P2126), tryptophan (Sigma, T0254), tyrosine (Sigma, T3754), were individually dissolved in MilliQ H_2_O at a concentration of 10 mM. 5M NaOH was added dropwise to the guanine solution until it fully dissolved. The deoxyribonucleotides dATP, dCTP, dGTP, dTTP (Sigma-Aldrich, DNTP100) were received as PCR-grade 100 mM aqueous solutions, the ribonucleotide UTP (Sigma-Aldrich, T25) was received as a powder that was subsequently dissolved in MilliQ H_2_O to a final concentration of 100 mM. Custom DNA/RNA strands were ordered (Sigma-Aldrich, VC00021 and VC40001) with the following single-strand 10mer sequences: DNA-A:5′-AAAAAAAAAA-3′, DNA-C: 5′-CCCCCCCCCC-3′, DNA-G: 5′-GGGGGGGGGG-3′, DNA-T: 5′-TTTTTTTTTT-3′, RNA-U: 5′-UUUUUUUUUU-3′. One 19 unit ssDNA strand, 5′-CAATTGTACTAGCCGGATC-3′, was designed to incorporate every possible base-pair combination without forming secondary structures, as assessed using the NUPACK analysis on-line tool^1^. All oligomers were received as 100 μM solutions. All solutions were diluted 1:1 with a 100 mM aqueous solution of Na_2_SO_4_, as an internal standard, and 50 μL of solution was dropped onto an Al wafer immediately prior to measurement. DUV Raman measurements were completed within 20 min of deposition to minimize the impact of evaporation.

### Artificial Mixture

A mixture of molecular standards was prepared according to the relative concentrations of the various major aromatic residues in *E. coli* undergoing rapid division with a doubling time of 40 min (see Table 3). The numbers of residues per cell were calculated from macromolecular composition data adapted by Milo et al. (2010) from the reports of Nierlich (1972), Neidhardt et al. (1990), and Neidhardt (1996), the proteome database from Kozlowski (2017), and the metabolite pool reported by Bennett et al. (2009). Because macromolecular nucleic acids represent such a large proportion of nucleobase residues, in order to accurately represent the composition of the cell, the DNA/RNA standards were used for the A, C, G, T, and U residues of nucleic acid, while dATP, dCTP, dGTP, dTTP, and UTP were used for the A, C, G, T, and U-containing free nucleotides. Phe, Trp, and Tyr were used for their equivalent residues in both macromolecular protein and the free metabolite pool. The total concentration of the mixture was 1.00 mM, equating to 0.26 fg per 0.9 μm^3^. Although this is a significantly lower total concentration of aromatic residues compared to that of the cell, a spectrum with a signal-to-noise ratio of 186:1 was still obtained. The spectrum exhibits the same major peaks as the cell spectrum, with the notable exception of a much more intense peak around ∼1600 cm^-1^ and a generally lower intensity for the minor peak regions (<1200 cm^-1^ and in between the major peaks).

### DUV Raman

MOBIUS (Mineralogy and Organic Based Investigations with UV Spectroscopy), A custom DUV resonance Raman spectrometer at the NASA Jet Propulsion Laboratory, was used for all measurements. MOBIUS uses a 248.6 nm NeCu pulsed laser (Photon Systems, Inc.) reflected off of a 248 nm RazorEdge ultra-steep long-pass edge filter (Semrock, Inc.) and focused onto the sample through a DUV chromatically corrected objective lens with a numerical aperture of 0.13 (ThorLabs LMU-5x-UVB). Raman-scattered photons were collected using 180° backscatter geometry, a Horiba 550i spectrometer, and a Horiba Symphony e2v 42-10 CCD liquid nitrogen cooled (-140°C) detector. Based on a 550 mm focal length, a slit width of 250 μm, and a grating groove density of 1800 lines/mm, the spectral accuracy was 3.8 cm^-1^ and the true spectral resolution (minimum peak-to-peak separation for distinguishing overlapping peaks) was ∼25 cm^-1^. A laser spot diameter of ∼75 μm had an energy at the sample of 0.8–1.2 uJ/pulse and a pulse width of 40 μs for cell measurements integrated over a total of 1200 pulses per point in order to minimize photodamage to the cell (Taguchi et al., 2011). The laser was replaced prior to measurements of the molecular standards, and adjusted to maintain a consistent output energy of 0.8–1.2 μJ per pulse. A total of 25 points in a 5 × 5 array were acquired for each sample. Prior to sample data collection, calibration was achieved by validating the position of the secondary laser line at 252.93 nm (McNeil et al., 1978)and at zero-order reflection. Resulting spectra were corrected for laser intensity variability using a normalized laser intensity correction factor, which represents the relative laser intensity during data acquisition. Cosmic rays were identified as outliers in the distribution of intensity values in each wavelength channel (Uckert and Bhartia, 2019) and replaced by the value of adjacent points. Downstream processing was completed using a combination of the R package HyperSpec and in-house python scripts utilizing SciPy (Jones et al., 2001) and LMFIT (Newville et al., 2014). Raman shifts were recalibrated using the atmospheric N_2_ peak to a standard peak position of 2330 cm^-1^ (Burris et al., 1992). The spectra were then sectioned to focus on the ‘organic fingerprint range’ between ∼800 and 1800 cm^-1^ (Zhu et al., 2011) and least-squares regression used to subtract a linear background. All steps are visualized in Supplementary Figure S2. Spectra in each sample were averaged and all cellular samples were intensity normalized relative to each other to the mean intensity of the guanine peak at ∼1460 cm^-1^. Cell spectra were fitted by scalable linear combination of individual molecular standard spectra, based on non-linear least-squares regression of all points between 800 and 1800 cm^-1^, which was done using the built-in functionality of python and the LMFIT package (Newville et al., 2014).

## Results

### Molecular Standards

**Figure 2:**
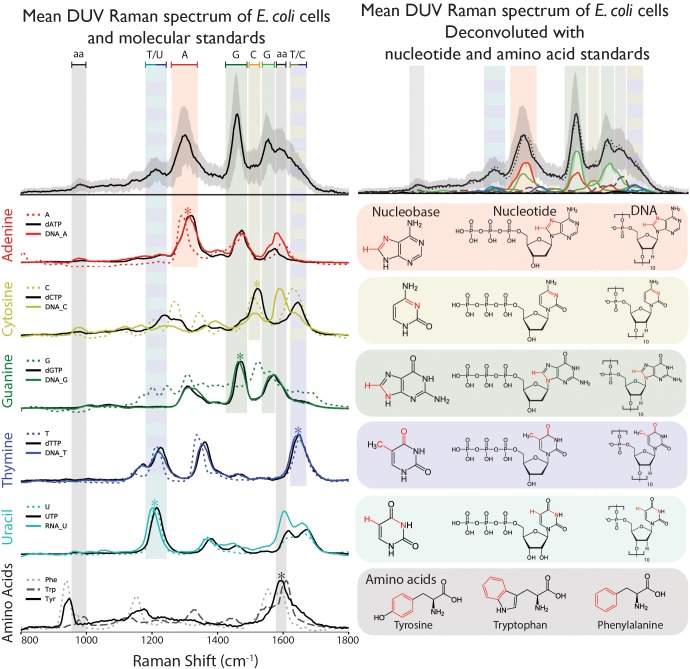
**(Top left)** Raman spectrum of *E. coli* taken under DUV (248.6 nm) excitation, with peaks assigned according to the dominant peaks of the nucleobases A, C, G, T, U, and the three aromatic amino acids (aa) Phe, Trp, and Tyr. **(Top right)** Deconvolution of the *E. coli* spectrum using the nucleotide and amino acid spectra, indicating relative contribution of different molecules to each peak. Raman spectra and chemical structures for the three sets of standards: nucleobases, nucleotides, and DNA/RNA, as well as the amino acids. Asterisks denote the dominant vibrational mode highlighted in red in the corresponding molecular structure.

Eight aromatic molecules: five nucleobases (adenine, A, cytosine, C, guanine, G, thymine, T, and uracil, U) and three amino acids (phenylalanine, Phe, tryptophan, Trp, and tyrosine, Tyr) that are known to contribute to the observed vibrational modes in the cellular spectrum were measured separately in solution. The nucleobases, which contribute the majority of Raman scattering at our chosen excitation wavelength, were measured in multiple forms of increasing structural complexity: simple nucleobases, deoxyribose/ribose nucleotide triphosphates, and single-stranded DNA/RNA 10-base oligomers containing mono-nucleotides of A, C, G, or T. As shown in Figure 2, each component exhibits a unique DUV Raman spectrum dominated by the resonant vibrational modes of its aromatic rings, with major peak positions and mode assignments presented in Table 1. For the simple nucleobases A, C, G, T and U, the dominant modes were 1291, 1512, 1440, 1647, and 1210 cm^-1^, respectively, that have been previously assigned to coupled vibrations of multiple bonds on each aromatic moiety (see Table 1) along with additional strong modes and several minor modes across the 800–1800 cm^-1^ range (see Supplementary Table S1 for assignments). The amino acids Phe, Trp, and Tyr exhibit similar spectra to one another dominated by the ring-stretching mode of the aromatic moiety at ∼1600 cm^-1^, in all three amino acids (Jenkins et al., 2005).

**Table 1:** Dominant peak positions (in cm^-1^) of the molecular standards and their assignments to vibrations of specific atomic bonds, categorized as either stretching (s), or bending (b) modes (Wen et al., 1997; Wen and Thomas, 1998).

Nucleobase	Simple NB	dNTP	DNA/RNA	Assignment
A	1291	1322	1314	C8H (b), C8N7 (s)
C	1512	1523	1508	N3C4 (s)
G	1440	1474	1462	C8H (b), N9C8 (s)
T	1647	1651	1634	C4 = O (s), C5C7 (s)
U	1210	1214	1203	C5H (b), N3C4 (s)

**Amino acids**	**Phe**	**Trp**	**Tyr**	

	1595	1610	1591	Benzene (s)

The nucleotides exhibit similar spectra to their respective nucleobases, though the frequencies and relative intensities of many peaks are altered by the addition of the ribose triphosphate. Specifically, the dominant modes in the nucleotide spectra are shifted to higher frequencies: adenine by 31 cm^-1^, cytosine by 12 cm^-1^, guanine by 34 cm^-1^, thymine and uracil by 4 cm^-1^. In most cases the number of peaks is unchanged, with the exception of dCTP, which exhibits fewer peaks than cytosine in the 1200–1400 cm^-1^ region. The spectra of the single-stranded DNA/RNA standards, each containing 10 units of a particular base, exhibit relatively slight spectral changes over the nucleotides: dominant peaks were consistently shifted to lower frequencies by 2–10 cm^-1^, with some alterations in relative intensities with respect to other modes. The most significant exception was the DNA-C 10-mer, which exhibits a strong mode (at ∼1574 cm^-1^) not previously observed directly in either cytosine or dCTP.

### Cellular Spectra

The spectrum of fixed dry cells was functionally identical to that of a fixed cell suspension (see Supplementary Figure S3) but provided better signal and a detectable N_2_ peak for reliable calibration. There were no differences between the spectra of each replicate (Supplementary Figure S4) and replicate A was used for further analysis. The DUV Raman spectrum of *E. coli* cells is dominated by peaks that are attributed to specific molecules based on comparison to their dominant vibrational modes: the nucleobases adenine, guanine, cytosine, and thymine, and the AAAs tyrosine, tryptophan, and phenylalanine (Figure 2). The predominance of these molecules in the DUV Raman spectrum can be explained by the resonant enhancement of their aromatic ring vibrations, which occurs when the excitation energy is comparable to the π-π^∗^ electronic transition localized on the aromatic ring. The molecular vibration of atmospheric N_2_ and O_2_ at ∼2331 and ∼1550 cm^-1^, respectively, were quantified and subtracted from the spectrum prior to further analysis. The cellular spectrum is characterized by three major peaks centered at ∼1310, ∼1470, and ∼1600 cm^-1^, derived from vibrational modes in adenine, guanine, and a combined peak from the overlapping vibrational modes of guanine and the AAAs. Peaks were assigned to specific vibrations of each molecule according to the literature (Wen et al., 1997): the dominant marker peak for adenine is assigned to a coupled vibration of C8N7 stretching, and C8H bending is apparent between 1220 and 1350 cm^-1^, with an observed peak center at 1305 cm^-1^. The characteristic ∼1450 cm^-1^ guanine peak is assigned to C8H bending/N7C8 stretching and is the sharpest, best-defined peak in the majority of spectra, centered at 1457 cm^-1^. The 1490–1650 cm^-1^ region is characterized by two peaks: a composite peak at 1540–1555 cm^-1^ due to overlapping modes of N3C4 stretching and C5C4/C4N3 stretching in guanine, and a ∼1600 cm^-1^ peak due to in-plane ring stretching of the AAAs. The broad, asymmetric tail of the 1600 cm^-1^ peak toward 1700 cm^-1^ indicates the presence of further vibrational modes that cannot be clearly defined due to their overlap, but are reported to include contributions from thymine, cytosine and guanine (Wen and Thomas, 1998). Finally, there is a small peak at ∼1750 cm^-1^ that is not assigned to any molecular vibration and is attributed to a secondary NeCu laser emission line reflected off the Al wafer used as a sample substrate. Secondary peaks apparent in the spectrum can be ascribed to vibrational modes in cytosine, thymine, and uracil. A peak centered at ∼1175 cm^-1^ is due to an undefined vibrational mode in thymine, ∼1200 cm^-1^ to a coupled C5H bending and N3C4 stretching vibration in uracil and ∼1510 cm^-1^ to N3C4 stretching in cytosine. A shoulder around 1350 cm^-1^ on the main adenine peak is due to C2H and N9C8 stretching in cytosine. A minor peak just below 1000 cm^-1^ is due to symmetric ring stretching in phenylalanine and tyrosine.

### Deconvolution

The deconvolution of the cell spectrum and of the artificial mixture spectrum were done with three different sets of standards, reflecting the increasing structural complexity of the nucleobases, with the AAAs represented by the same Phe, Trp, and Tyr spectra in all cases. The relative integrated intensities of each component in each fit were obtained based on their respective fitting coefficients, with uncertainties derived from the coefficient’s standard error and the standard deviation of the spectrum. In all cases, the five nucleic acids represented the majority of Raman intensity across the cellular spectrum. The overall goodness of each fit was expressed numerically by its chi-squared (χ^2^) value, constructed from the sum of the square of the fit’s residuals. The absolute values of χ^2^ obtained were large, due to the fit having 253 degrees of freedom, with the poorest fit having an χ^2^ of 320 and the best fit having an χ^2^ of 49.

## Discussion

### Biological Patterns as Revealed by DUV Raman Spectroscopy

The three AAAs Phe, Trp, and Tyr exhibit DUV Raman spectra with unique patterns of minor vibrational modes across the 800–1800 cm^-1^ range (see Figure 2), but share a common dominant mode at 1600 cm^-1^ that makes them difficult to distinguish in mixtures but can be treated as indicative of the presence of AAAs. It is of importance to note that AAAs have not been identified in extraterrestrial material. Tryptophan, specifically, has the highest free energy of formation (Amend and Shock, 1998), as such the observation of AAAs is potentially indicative of biosynthetic pathways. In contrast to the AAAs, the five nucleobases A, C, G, T, and U exhibit unique Raman spectra dominated by different vibrational modes at distinct frequencies (Figure 2). The pattern of peak intensities was not consistent between molecules, beyond being assigned to aromatic modes, due to the dependence of Raman scattering cross-sections and vibrational frequencies on the exact structure of each molecule. In general, the most intense peaks of the five nucleobases are sufficiently separated in frequency that they can be considered specific markers for each base. Furthermore, individual spectra are sensitive to increasing structural complexity; there are significant spectral changes between the nucleobases and their respective nucleotides, consisting primarily of shifts in peak position and the suppression of various minor peaks. Changes in vibrational frequency, such as the ∼31 cm^-1^ shift in the major C8H/C8N7 mode between adenine and dATP, can be attributed to a redistribution of π-electron density on the adenine’s aromatic purine moiety upon addition of the deoxyribose triphosphate unit. Changes in the relative intensity of various modes, to the point of peaks disappearing for cytosine and guanine, is likely due to vibrational coupling between in-plane vibrations of the aromatic and ribose moieties, suppressing some Raman peaks while enhancing others. This coupling has been reported previously based on the sensitivity of DUV resonant aromatic vibrational frequencies to selective deuteration of the ribose unit (Toyama et al., 1993). The precise impact varies from nucleobase to nucleobase, due to the different structures of their aromatic moieties leading to different degrees of coupling, with some exhibiting more significant shifts in frequency or fewer suppressed modes, e.g., adenine and cytosine respectively.

Further increase in structural complexity from nucleotides to DNA is expected to have a small but definite impact on the Raman spectra of the various nucleobases, due to π-π stacking between neighboring nucleobases altering the electron density on their aromatic moieties and therefore changing their vibrational properties. By using relatively short oligomers of single-stranded DNA, each nucleobase can be assessed individually, without hydrogen bonding interactions caused by Watson–Crick pairing or larger scale structure. When compared in Figure 2, we observe that the majority of each nucleotide’s Raman peaks appear in the DNA spectrum as well. Minor changes were observed, e.g., a slight broadening of the bimodal ∼1300 cm^-1^ guanine peak, or a small (∼8 cm^-1^) down-shift of the thymine peaks at ∼1200 and 1300 cm^-1^. There were also more significant effects: the relative intensity of the ∼1550 cm^-1^ adenine peak increases between dATP and the DNA-A 10-mer; the spectrum of the DNA-C 10-mer is dominated by a mode at ∼1574 cm^-1^ that was a hidden peak in cytosine and dCTP; and the ∼1600 and ∼1650 cm^-1^ modes of uracil both increase in relative intensity. We attribute all of these spectral changes to the π-π stacking of neighboring nucleobases within DNA, which are extremely unlikely to occur between free nucleotides in solution at the concentrations being considered. The effect of close interactions between aromatic π systems is well-known, producing easily measurable, if difficult to predict, changes in vibrational frequency and relative peak intensities under resonant excitation (Milani et al., 2007). It is not immediately clear if these minor changes make the DNA/RNA standards more representative of the cell spectrum, and a more thorough method of comparison is required.

The DUV Raman spectrum of *E. coli* consists of several peaks all of which can be attributed to vibrational modes in at least one of molecular standards used based on comparisons with the dominant vibrational modes of those molecules. The overall Raman spectrum of the cell may be considered a composite of the Raman spectra of all the components of the cell, weighted by the number of each molecule and their relative Raman cross-sections. Two well-defined peaks at ∼1310 and ∼1470 cm^-1^ are consistent with vibrations in adenine and guanine respectively, with several more minor peaks and overlapping modes assigned in Figure 2. The Raman spectrum can be modulated by reduction (hypochromism) or increase (hyperchromism) in Raman cross-section due to intermolecular interactions, such as π-π base stacking and Watson–Crick pairing in DNA, as has been reported for both nucleobase and amino acid peaks in DUV Raman spectra of biological matter (Wen et al., 1997). Specific hypochromism in nucleic acids observed as a decrease in Raman scattering of DNA bases when stacked in DNA has be previously reported (Bolton and Weiss, 1962). Identification of the constituent DUV resonant molecules is still possible despite these phenomena; however, as acknowledged earlier, even the most diagnostic peaks are complicated by significant degrees of overlap with the minor vibrational modes of the other components, and assigning peaks to only the apparent dominant modes without accounting for composition may lead to incorrect interpretation of spectra.

**Figure 3:**
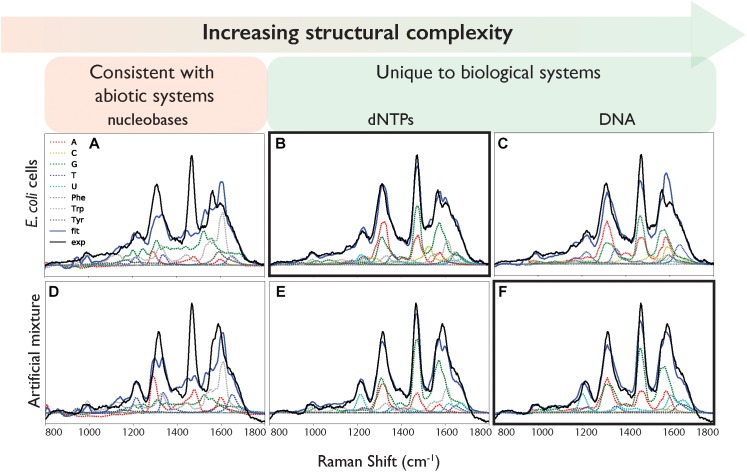
Deconvolution of the DUV Raman spectra of **(A–C)** *E. coli* and **(D–F)** the artificial mixture, using **(A,D)** nucleobase and amino acid spectra, **(B,E)** using nucleotide and amino acid spectra, **(C,F)** using ssDNA and amino acid spectra. Exp: the calibrated mean experimental data. Fit: the fit result of the linear combination of components.

Spectral deconvolution allows for consistent identification of the components present within a complex, mixed spectrum dominated by several aromatic molecules. Deconvolution must be informed by prior measurements of standard spectra for a set of known molecules that are representative of the total system. With three sets of molecular standards of increasing structural complexity, we deconvolute the spectrum of *E. coli* in order to establish the composition of the cellular spectrum, and the minimum degree of complexity required to distinguish the cell from its most simplistic parts. Deconvolution was done by scalable linear combination of the appropriate molecular standard spectra (including the five nucleobases and three AAAs; expressed in Eq. 1), varying only their respective fitting coefficients (i.e., their relative intensities) to minimize the residual between *I_fit_* and *I_cell_*, with the results presented in Figure 3.

(1) $$I_{\text{fit}}\;=\;a\;*\;Adenine\;+\;c\;*\;Cytosine\;+\;g\;*\;Guanine\;+\;t\;*\;Thymine\;+\;u\;*\;Uracil\;+\;f\;*\;Phenylalanine\;+\;w\;*\;Tryptophan\;+\;y\;*\;Tyrosine$$

The “goodness of fit,” defined by the sum of the square of the residual, varied depending on the structural complexity of the nucleobase components (Figure 3 and Table 2). When the simple nucleobases are used as components for deconvolution, the main adenine mode at ∼1310 cm^-1^ is adequately described, but the rest of the spectrum is fit rather poorly: failing to account for major modes including the guanine peaks at ∼1469 and ∼1572 cm^-1^; overestimating the intensity of the amino acid peak at ∼1600 cm^-1^; and underestimating the minor contribution of weak modes around 900–1100 cm^-1^ (see Figure 3A). The failure to account for the major guanine peak at ∼1469 cm^-1^ can be attributed to the lack of any equivalent mode in the standard guanine spectrum, with the closest guanine mode some ∼29 cm^-1^ lower in frequency and much less well-defined. The over-estimation of intensity at ∼1600 cm^-1^ is mostly due to a contribution from adenine in that region, even though that peak is typically associated with the amino acids. It was not possible to comment on the relative contributions of the three AAAs as the fitted coefficients of Phe, Trp, and Tyr were significantly cross-correlated (Supplementary Table S2) such that their intensities cannot be considered separately and must be treated as a single, total ‘aromatic amino acid’ component that was significantly overestimated in the nucleobase fit.

**Table 2:** Intensities for each component of the nucleic acid + aromatic amino acid (AAA) deconvolutions of *E. coli* and the artificial mixture, fitted using standards of varying degrees of nucleic acid structural complexity.

Fit:	Adenine	Cytosine	Guanine	Thymine	Uracil	Phe	Trp	Tyr
**Cell**								
Nucleobase + AAAs (χ^2^ = 320)	0.10 ± 0.04	0.09 ± 0.06	0.44 ± 0.07	0.08 ± 0.04	0.06 ± 0.02	0.08 ± 0.03	0.00 ± 0.00	0.03 ± 0.02
Nucleotide + AAAs (χ^2^ = 49)	0.23 ± 0.05	0.14 ± 0.03	0.34 ± 0.05	0.06 ± 0.01	0.06 ± 0.01	0.09 ± 0.03	0.02 ± 0.01	0.02 ± 0.01
DNA + AAAs (χ^2^ = 140)	0.34 ± 0.12	0.03 ± 0.01	0.32 ± 0.10	0.11 ± 0.03	0.11 ± 0.04	0.02 ± 0.02	0.01 ± 0.00	0.00 ± 0.00
**Artificial mixture**								
Nucleobase + AAAs (χ^2^ = 217)	0.15 ± 0.07	0.00 ± 0.00	0.22 ± 0.04	0.12 ± 0.06	0.00 ± 0.00	0.00 ± 0.00	0.34 ± 0.11	0.06 ± 0.02
Nucleotide + AAAs (χ^2^ = 62)	0.18 ± 0.04	0.00 ± 0.00	0.50 ± 0.08	0.06 ± 0.01	0.12 ± 0.02	0.00 ± 0.00	0.17 ± 0.06	0.00 ± 0.00
DNA + AAAs (χ^2^ = 54)	0.27 ± 0.10	0.03 ± 0.01	0.44 ± 0.14	0.07 ± 0.02	0.16 ± 0.05	0.00 ± 0.00	0.05 ± 0.02	0.00 ± 0.00

*Intensities are integrated over 800–1800 cm-^*1*^ and relative to total intensity of the cell. Relative goodness of each fit is given by the value of chi-squared, i.e., the sum of the square of the residuals*.

When the cell deconvolution was run using the nucleotides as components, the fit immediately improved: the adenine/guanine peaks at ∼1300, ∼1450, and ∼1550 cm^-1^ are adequately represented by the adenine and guanine components in both peak intensity and shape (Figure 3B). The remaining residuals are more evenly distributed and while there are still the distinctively shaped residuals produced by deviations in vibrational frequency for certain peaks (Supplementary Figure S5), there is clearly much better correlation than with the nucleobases. We can conclude that the nucleotides are significantly more representative of the vibrational properties of their respective equivalents within the cell itself, compared to the nucleobases. The remaining minor deviations may be the result of the slight vibrational changes observed between nucleotides and the more structurally complex DNA bases, or evidence for the collective signal from non-DUV resonant components within the cell.

**Figure 4:**
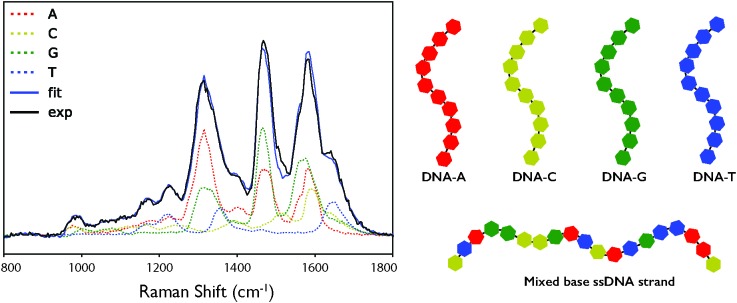
**(A)** Deconvolution of the DUV Raman spectrum for a single-stranded DNA sequence (5′-CAATTGTACTAGCCGGATC-3′) using the individual DNA and amino acid spectra. Exp: the calibrated mean experimental data. Fit: the fit result of the linear combination of components. **(B)** Schematic representation of the mononucleotide and mixed based ssDNA strands used as standards.

When the cell deconvolution was run using the DNA base standards, the result was a poorer fit than with the nucleotide standards (χ^2^ = 140 compared to 49). As shown by Figure 3C, there is a large error at ∼1550 cm^-1^ that was not present in the nucleotide fit. This corresponds to the C4N3/C5C4 mode of adenine, which exhibited a much larger relative intensity in the DNA-A 10-mer vs. dATP, along with a smaller contribution from the additional mode of the DNA-C 10-mer that was not present in dCTP. Further deviation occurs around the dominant adenine and guanine peaks of the cellular spectrum, at ∼1300 and 1450 cm^-1^, due to the broader modes of the DNA-A and DNA-G 10-mers spectra vs. dATP and dGTP. The deviation cannot be easily ascribed to the necessarily artificial single-base sequences of these standards: when a mixed DNA 19-mer containing all four bases (with a 5:5:4:5 ratio of A:C:G:T) was measured, it had a spectrum that was a clearly linear combination of the relevant DNA base standards (Figure 4). This shows that there are no spectral shifts or changes resulting from neighbor-neighbor interactions and secondary structure, though we cannot rule out the possible effects of tertiary structure as the mixed DNA sequence was specially chosen to limit pairing interactions.

Fundamentally, the cell spectrum can be adequately described by nucleotide and AAA spectra. The nucleotides must be sufficiently complex to represent the nucleic acid component, in a manner that the simpler nucleobases were not. It is of note that nucleotides have not been observed to occur in abiotic systems in contrast to nucleobases. The detection of these component molecules in combination, reflecting the complexity in chemical structure and composition of the cell, can therefore be considered a meaningful biosignature detectable by DUV Raman spectroscopy.

### Macromolecular Composition of the Cell

Deconvolution of the cellular Raman spectrum may provide a first approximation of cellular composition, modulated by differences in Raman cross-section between detectable components. The fact that the dNTP standards provide a better fit than the DNA standards is surprising, considering that nucleic acids account for the majority of nucleobases in the cell (Neidhardt et al., 1990). In order to investigate this phenomenon, we first must approximate the macromolecular composition of the cell and those components that are detectable by DUV Raman.

While it is well-established that the composition of the cell varies over time (Pahlow et al., 2015; Hlaing et al., 2016), the values given here are based on average *E. coli* cells during exponential growth and thus should be an acceptable first approximation of the composition of the cells that were measured using Raman spectroscopy in this study. The overall composition of an average *E. coli* cell was calculated based on adaptations of the values for the macromolecular composition of *E. coli* by Milo et al. (2010) from Neidhardt et al. (1990) and others, to reflect uncertainties for a cell growing exponentially at 37°C in aerobically balanced glucose minimal media with a doubling time of 40 min (Figure 1 and Supplementary Table S3). We expect that only the aromatic units will be resonantly enhanced by DUV excitation, and we approximate the aromatic-containing components with the eight molecules that are known to contribute to the DUV Raman spectrum. Bennett et al. (2009) used mass spectrometry to quantify 103 metabolites within the cell and we approximate the DUV resonant fraction as those molecules that contain at least one of the eight aromatic moieties (see Supplementary Table S3). Given a total wet mass of ∼1000 fg and a volume of ∼0.9 μm^3^ per cell (Milo et al., 2010), we calculated the number of DUV resonant residues present in each group. Protein accounts for 165 fg per cell, with Phe, Trp, and Tyr accounting for 7% of residues (Kozlowski, 2017) equating to 65 million aromatic residues per cell. Assuming a rapidly dividing cell contains on average 2 genomes accounting for nested chromosomal replication, DNA comprises ∼9 fg, and RNA ∼60 fg (Milo et al., 2010), and based on known A/C/G/T and A/C/G/U mole ratios, the nucleic acids contain 16.6 and 106 million nucleobases per cell respectively (Nierlich, 1972; Blattner et al., 1997). Nucleobases in the metabolome total 36 million per cell, primarily in molecules containing adenine and uracil (Table 3; Bennett et al., 2009). The prevalence of RNA over DNA is expected for a cell undergoing rapid growth (Bremer and Dennis, 2008), and we find that 77% of all nucleobases within the cell are sequestered in nucleic acids, with only 23% represented as free metabolites. The AAAs are a very minor fraction of the metabolome, totaling less than 40,000 molecules per cell, meaning that 99.4% of all AAA residues occur in proteins. In total, we estimate there are roughly 224 million DUV resonant aromatic molecules per cell, 71% of which are nucleotides and 29% are amino acids, accounting for a total of 12% of cell mass. With water accounting for ∼64% of cellular mass, the remaining ∼21% is expected to consist of molecules that lack a considerable DUV Raman cross-section, their collective Raman scattering and interactions with DUV resonant molecules may still contribute substantially to measured Raman intensity, and it is important to consider what effect they may have on the overall spectrum.

**Table 3:** Approximate composition of DUV resonant macromolecular components in an average cell of *E. coli* during exponential growth with a doubling time of 40 min, based on a cellular volume of 0.9 μm^3^ (Neidhardt et al., 1990).

				Aromatic
	% cell	Weight per		units per
Macromolecule	mass	cell (fg)	% mol	cell (x10^6^)
**Protein ^1^**	**16**.**5**	**165**		**65**.**2**
Phe			3.46	31.8
Trp			1.06	9.74
Tyr			2.58	23.6
**RNA ^2^**	**6**	**60**		**106**
A			24.8	26.2
C			21.8	23.0
G			32.4	34.2
U			21.0	22.2
**DNA ^3^**	**0**.**9**	**9**		**16**.**6**
A			24.5	4.07
C			24.6	4.08
G			26.2	4.35
T			24.7	4.10
**Metabolites ^4^**	**7**	**70**	**Concentration (mM)**	**36**.**4**
A			16.0	10.7
C			2.75	1.84
G			5.85	3.91
T			4.98	3.33
U			22.7	15.2
Phe			2.00	0.012
Trp			1.33	0.0008
Tyr			3.22	0.019
**Other**	**5**.**7**	**57**		
**Water**	**63**.**9**	**639**		
**Total**	**100**	**1000**		**224**

*(1) Calculated based on 3 × 10^6^ proteins per cell (Neidhardt et al., 1990), cumulative weight derived from Neidhardt et al. (1990), amino acid abundance from the proteome-pl database (Kozlowski, 2017), and the molecular mass of each amino acid. (2) Calculated using the cumulative weight derived from Neidhardt et al. (1990), the RNA composition reported by Nierlich (1972) and the individual masses of the ribonucleotide monophosphates. (3) Calculated using the cumulative weight derived from Neidhardt et al. (1990), the DNA composition from Blattner et al. (1997) and the individual masses of the deoxyribonucleotide monophosphates. (4) Calculated from concentrations of 103 free metabolites given by Bennett et al. (2009), grouped according to constituent aromatic unit. Bold values indicate totals for each macromolecule class*.

### The Cell Approximated by the Sum of Its Parts

With the evidence available so far, it appears that there is clearly some effect at play that distorts Raman intensities beyond what would be expected solely from composition, leading to a spectrum dominated by a relatively minor fraction of the population of DUV resonant molecules within the cell. To assess if this variation is a function of biological structure, we can ask if the cell is truly more than the sum of its parts, i.e., is the observed spectrum unique to the cell and distinguishable from an artificial mixture containing the same components. If true, then the residual of the cell vs. the components may be treated as a biosignature in of itself, being a summation of the collective structure and complexity within the cell that, by definition, does not exist in the artificial mixture. To this end, the DUV resonant components were mixed according to their relative concentrations within the cell, derived from Table 3 (see Supplementary Table S4 for a detailed breakdown of approximations), and a Raman spectrum obtained of the mixture.

**Figure 5:**
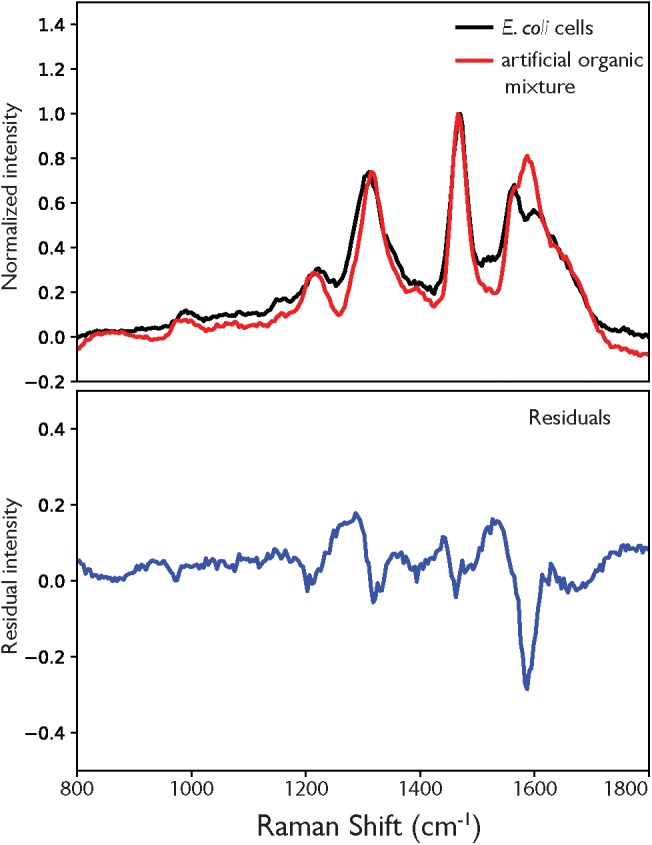
**(A)** Comparison of the DUV Raman spectra for *E. coli* and a mixture of abiotic molecules with composition representative of an *E. coli* cell, normalized to the guanine peak at 1469 cm^-1^, with residual in blue **(B)**.

As shown in Figure 5, the artificial mixture exhibits a similar spectrum to that of the cell, recreating the positions and relative intensities of the major peaks with reasonable accuracy, demonstrating that the mixture has effectivity recreated the relative composition (and spectral contributions) of the cell in terms of its most DUV resonant components. The largest single deviation is the additional peak at ∼1590 cm^-1^, which at first appears to relate to the AAA component but does not perfectly align with the dominant amino acid mode at 1600 cm^-1^. When the spectrum from the artificial mixture was deconvoluted, the best fit was obtained using DNA standards (see Figure 3D–F and Supplementary Figure S6) with the extra peak described not by any of the amino acids but by the DNA-A 10-mer, namely the bimodal vibration at ∼1583 cm^-1^. Aside from the erroneous extra peak, the difference between cellular and abiotic spectra consisted mainly of additional background signal across the organic fingerprint region (800–1800 cm^-1^) that was apparent in the cell spectrum but not in the mixture, and accounts for 16% of total intensity across the range in question. This background cannot be attributed to molecular fluorescence, as the frequencies of Raman-scattered light under DUV excitation are significantly higher than that of photo-luminescence, nor is it an artifact of sample configuration as both spectra were measured of samples in the same conditions on the same substrate material, which does not contribute any signal in this range.

It is clear that there are distinctive and measurable spectral features that distinguish a cell from a simple mixture of its most DUV resonant components. There are three possible explanations for why the artificial mixture deviates from the cell: (1) the cumulative contribution of all the non-DUV resonant components of the cell that were not included, (2) the lack of tertiary structure for the nucleic acid components, and (3) the free metabolites are not easily represented by their equivalent dNTP/NTP nucleotide.

There is low intensity Raman scattering across the 800–1800 cm^-1^ range observed for the cell that is not apparent in the artificial mixture. This could not be attributed to fluorescence or other background effects, and may instead represent the total contribution from all non-resonant components that were not included in the mixture, but comprise approximately two thirds of the non-water mass of the cell. Considering the variety of species that group includes, such as non-AAAs, lipids and sugars, among others, the cumulative Raman scattering from their diverse vibrational modes should extend across the entire organic fingerprint region, with few distinguishable peaks. This is consistent with what we observe, as the residual (Figure 5B) exhibits no clearly defined peaks that are not assigned to a vibrational mode present in the DNA standards.

The artificial mixture was best fitted with the DNA standards (see Supplementary Figure S6 for residuals and residual distributions), while the cell was best fitted using the nucleotide standards. In the artificial mixture, nucleic acids were represented by a representative proportional mixture of 10-unit oligomers of each base while in the cell these molecules are often present in complex three-dimensional conformations. We suspect that this is due to differences in the relative Raman cross-sections of the nucleobases in the free molecule vs. the macromolecule: that either the free nucleotides produce stronger Raman scattering per aromatic unit than the same nucleotides in DNA/RNA, or that tertiary structure diminishes the Raman cross-section of the aromatic unit in the nucleic acid, reducing its effective intensity consistent with previous studies (Supplementary Figure S7; Bolton and Weiss, 1962). This may in part be due to chromosomal and RNA packing: over 80% of total RNA is tightly folded into ribosomes (Bremer and Dennis, 2008). We have noted that differences in Raman cross-section can lead to two standards giving different apparent intensities even at the same concentration: this is illustrated by a DNA-mix 19-mer, which has a known A, C, G, T molar composition of 26, 26, 21, and 26% but integrated intensities from fitting were 37, 17, 33, and 12% respectively, indicating that per molecule the purines produce greater Raman scattering than the pyrimidines. It is probable that the introduction of tertiary structure, where every nucleobase is surrounded by other aromatic molecules and proteins, diminishes the Raman cross-section of the aromatic ring such that the nucleic acids contribute less intensity than expected given their proportion within the cell. However, it does empirically demonstrate that the DUV Raman spectrum of the cell is sensitive to this larger-scale structure that may distinguish it from its mere components.

With further work, deconvoluting the cellular spectrum into its components could be a potentially useful tool for studying terrestrial cellular activity as well as detecting biosignatures. Such analysis would require a thorough understanding of the Raman activities of the component molecules, based on the collection of calibration curves to correlate Raman intensities to concentrations. With that information, it should be possible to derive the Voronoi plot of cellular composition in Figure 1 from that of the Raman deconvolution. Providing the ability to spectroscopically measure changes in the composition of the cell, based on changes in the deconvolution of the Raman spectrum, would allow investigation into RNA expression and protein production as a function of cell growth rate and species differentiation based on comparisons of genome GC content and differential protein expression. However, obtaining the relevant calibration curves is not a trivial process for such a complex system as an entire cell: additional work must be done to establish the obfuscating factors that may further modulate intensities for these components in this environment, including component–component interactions, before we can employ quantitative DUV Raman spectroscopy as a tool for studying microbiology at the cellular level. While the proprinquitous detection of complex aromatic molecules not expected to exist together at the observed concentrations spontaneously, constitutes a potential DUV Raman biosignature, the apparent sensitivity of the cellular spectrum to tertiary structure provides direct evidence of larger-scale structure and complexity that cannot exist in abiotic systems, strengthening the interpretation of biogenicity. It seems that, from a spectral perspective, the cell is indeed more than the sum of its parts.

Deep UV Raman spectroscopy has been selected as an instrument on the Mars 2020 rover in part due to its sensitivity and specificity for the detection of aromatic organic molecules (Beegle et al., 2015). We do not predict here the specific aromatic organics that may be detected on Mars nor do we attempt to approximate the mineral matrix in which they may be preserved. Rather we demonstrate the importance of molecular complexity to the interpretation of DUV Raman spectra of aromatic organic molecules fundamental to terrestrial life. The mechanisms that led to the structural organization of pre-biotic organic compounds into complex assemblages conferring the functions of energy transduction, replication, and information storage are currently unknown. It can be argued that the emergence of specifically structured functional complexity gave rise to molecular assemblages capable of preforming the functions that we associate with life. At a basic level, these processes harness free energy to predictably and systematically produce specific outcomes that without facilitation or catalysis by living systems would not be predicted to occur. A defining characteristic of life is to produce low probability outcomes reflected in characteristic enrichments of specific organic molecules (e.g., McKay, 2004; Des Marais et al., 2008; Des Marais, 2013; Mustard et al., 2013). For example Fischer-Tropsch-Type synthesis is hypothesized to account for α amino acids in carbonaceous chondrites leading to a thermodynamically driven distribution characterized by a decrease in abundance with increasing carbon chain length (Donnelly and Satterfield, 1989) as recorded in the organic inventory of amino acids in extraterrestrial samples (e.g., Pizzarello et al., 2006) whereas biogenic processes enrich thermodynamically costly, structurally complex, molecules such as ∼C17–C31 alkanes and aromatic rings (Lovelock, 1965; Scalan and Smith, 1970; Amend and Shock, 1998; Kuhn et al., 2010). Biosignatures reflect the persistence of these low probability outcomes, recording the mechanisms of energy capture and transduction into the unlikely emergence of complexity. It is established that simply identifying aromatic molecules does not constitute evidence of life. We show that molecular complexity is significant and the DUV Raman spectra of those molecules can be used to define a threshold for aromatic organic molecules uniquely associated with life. The premise of astrobiology relies on the assumption that the activity of living organisms will result in the formation of geochemical, molecular, and/or structural patterns that are both recognizable and distinguishable from the environment in which they formed and that their presence is statistically unachievable within a purely abiotic system (Cady et al., 2003; Des Marais et al., 2008; Summons et al., 2008; Mustard et al., 2013). While it is not expected that life beyond Earth would necessarily be comprised of the same subset of organic molecules, specificity and patterns indicative of biological complexity is thought to be a universal attribute of life (e.g., Summons et al., 2008). When searching for biosignatures beyond Earth it is imperative that these universal traits are interrogated (Des Marais, 2013). Here, we use DUV Raman spectroscopy to evaluate the increasing complexity of biomolecules and the ability of these individual components to deconvolve cellular spectra to illustrate the role of emergent molecular complexity in a cell as a fundamental component in biosignature detection.

## Conclusion

The deconvolution of the cellular *E. coli* Raman spectrum using molecular standards of increasing complexity has provided several valuable insights into the detection of biosignatures using DUV Raman spectroscopy. Firstly, this technique is capable of distinguishing between a mixture of aromatic molecules and a complex cell built from structured components, as demonstrated by the difference between the ‘best’ fit spectrum using simple nucleobases vs. nucleotides. This is important because although the simple nucleobases have been detected in abiotic environments such as meteorites and molecular nebulae, they do not constitute a biosignature in of themselves. Secondly, we have confirmed that we can differentiate a cell from DNA based on its spectra and that the resulting spectra cannot be explained simply by the spectral contribution of AAAs, but rather is primarily due to the intracellular pool of free nucleotides combined with the hypochromatism of nucleobases when stacked in nucleic acids. Third and finally, we have shown that nucleotides are of sufficient structural complexity to adequately describe cellular spectra, and that obtaining standard spectra of more complex molecules may not be necessary to identify biosignatures using Raman.

It is evident that an *E. coli* cell as described by its DUV Raman spectrum is more than the sum of its DUV resonant components. While the characteristic peaks in the cellular spectrum may be assigned by the dominant molecular vibrations of the DUV resonant components as a first approximation, it is clear that a specific combination of these components at a sufficient level of molecular complexity is required to adequately describe the cellular spectra by means of deconvolution. The observed cellular spectrum is a function of (1) the combined relative Raman cross-section of each component and; (2) the expression of that component within the cell. The former enables the selective investigation of a smaller, but still representative, subset of aromatic molecules by using DUV excitation. The cellular expression of these components is a function of billions of years of evolution selectively accumulating organic molecules, transferring a level of functional complexity reflected in a unique association of specific molecules not expected to have occurred by chance in a purely abiotic system. This study demonstrates the ability of DUV Raman spectroscopy to interrogate the nature of biological complexity and differentiate an organic signal from a definitively biological one.

## Author Contributions

HS designed the study with significant input from JH, LB, and RB. HS cultured the cells, prepared the cell samples, and collected the cellular Raman spectra. HS and JH designed the analytical pipeline and wrote the processing code. JH made the standard solutions, collected the standard Raman spectra, and finalized the Raman analyses. HS and JH wrote the manuscript with significant input from RB and LB. JA and VO contributed to discussion, feasibility, and text.

## Conflict of Interest Statement

The authors declare that the research was conducted in the absence of any commercial or financial relationships that could be construed as a potential conflict of interest.

## Footnotes

1 nupack.org

## Abbreviations

AAA: aromatic amino acid. In this paper, refers to phenylalanine, tryptophan, and tyrosine
dATP: deoxyribose adenosine triphosphate, a nucleotide of adenine
dCTP: deoxyribose cytidine triphosphate, a nucleotide of cytosine
dGTP: deoxyribose guanosine triphosphate, a nucleotide of guanine
dTTP: deoxyribose thymidine triphosphate, a nucleotide of thymine
DUV: deep ultraviolet, light with a wavelength ∼100–300 nm
MOBIUS: Mineralogy and Organic Based Investigations with UV Spectroscopy
Nucleobase: molecular derivatives of purine and pyrimidine. In this paper, refers to adenine, cytosine, guanine, thymine and uracil
Nucleotide: molecules containing a nucleobase, a ribose unit and a triphosphate group
SHERLOC: Scanning Habitable Environments with Raman and Luminescence for Organics and Chemicals
UTP: ribose uridine triphosphate, a nucleotide of uracil.
