# Supplementary material for: The Cell and the Sum of Its Parts: Patterns of Complexity in Biosignatures as Revealed by Deep UV Raman Spectroscopy
Source: Front Microbiol. 2019 May 14;10:679. doi: 10.3389/fmicb.2019.00679 (PMC6527968; doi:10.3389/fmicb.2019.00679)
Supplement: Supplementary file 1 [file Data_Sheet_1.docx]

**The cell and the sum of its parts: patterns of complexity in biosignatures as revealed by DUV Raman spectroscopy**

**Authors:** Sapers, Razzell Hollis, Bhartia, Beegle, Orphan, Amend

Supplementary Information


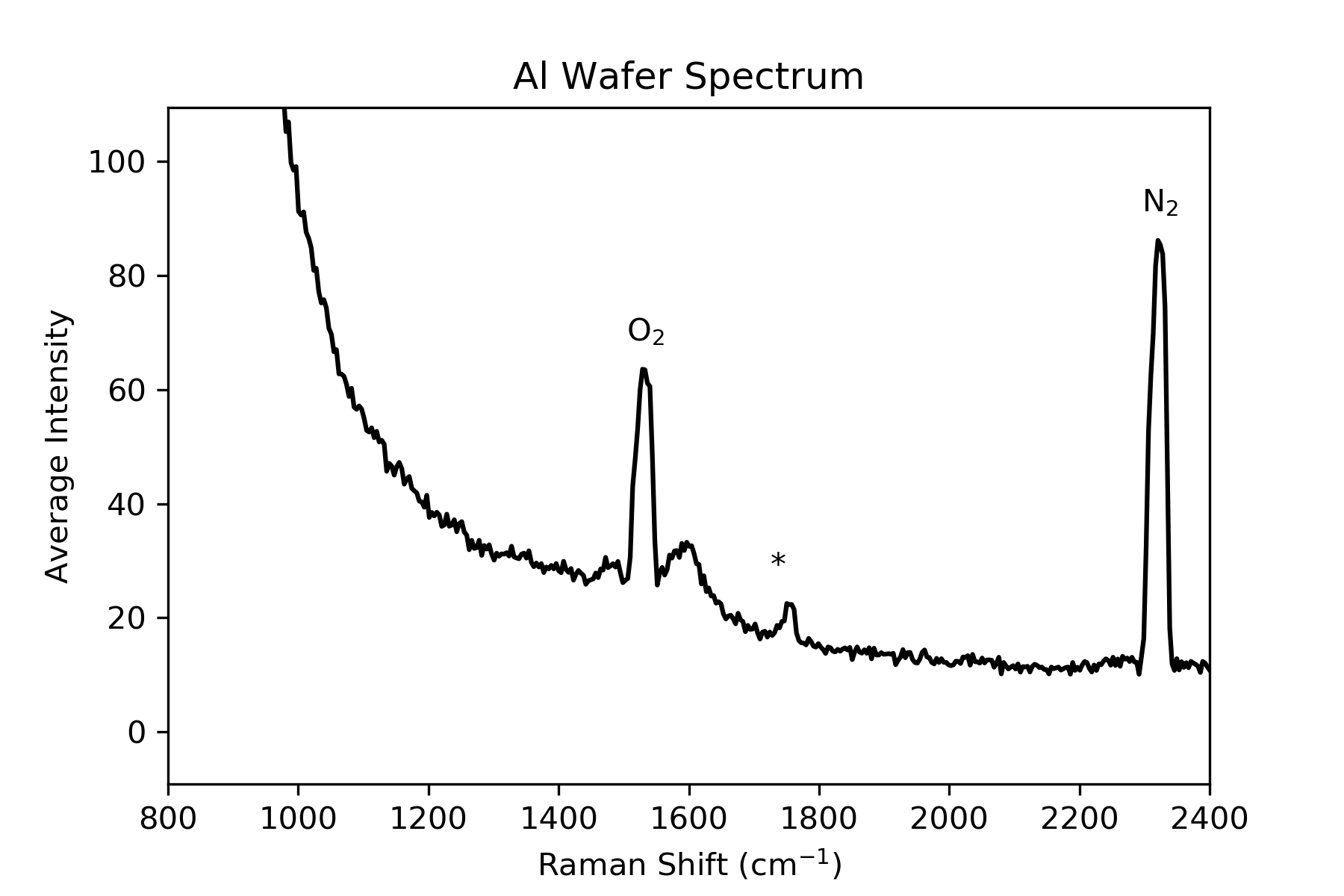


Figure S1: Raman spectrum of the Al wafer, averaged over 25 points with no further processing. No intrinsic peaks from the wafer are observed, only atmospheric N_2_ and O_2_ peaks, with satellite bands from rovibrational transitions of O_2_, and a minor laser line at 1750 cm^-1^ (*). Note that the intensities presented here are roughly an order of magnitude weaker than in the cellular and molecular standard spectra.


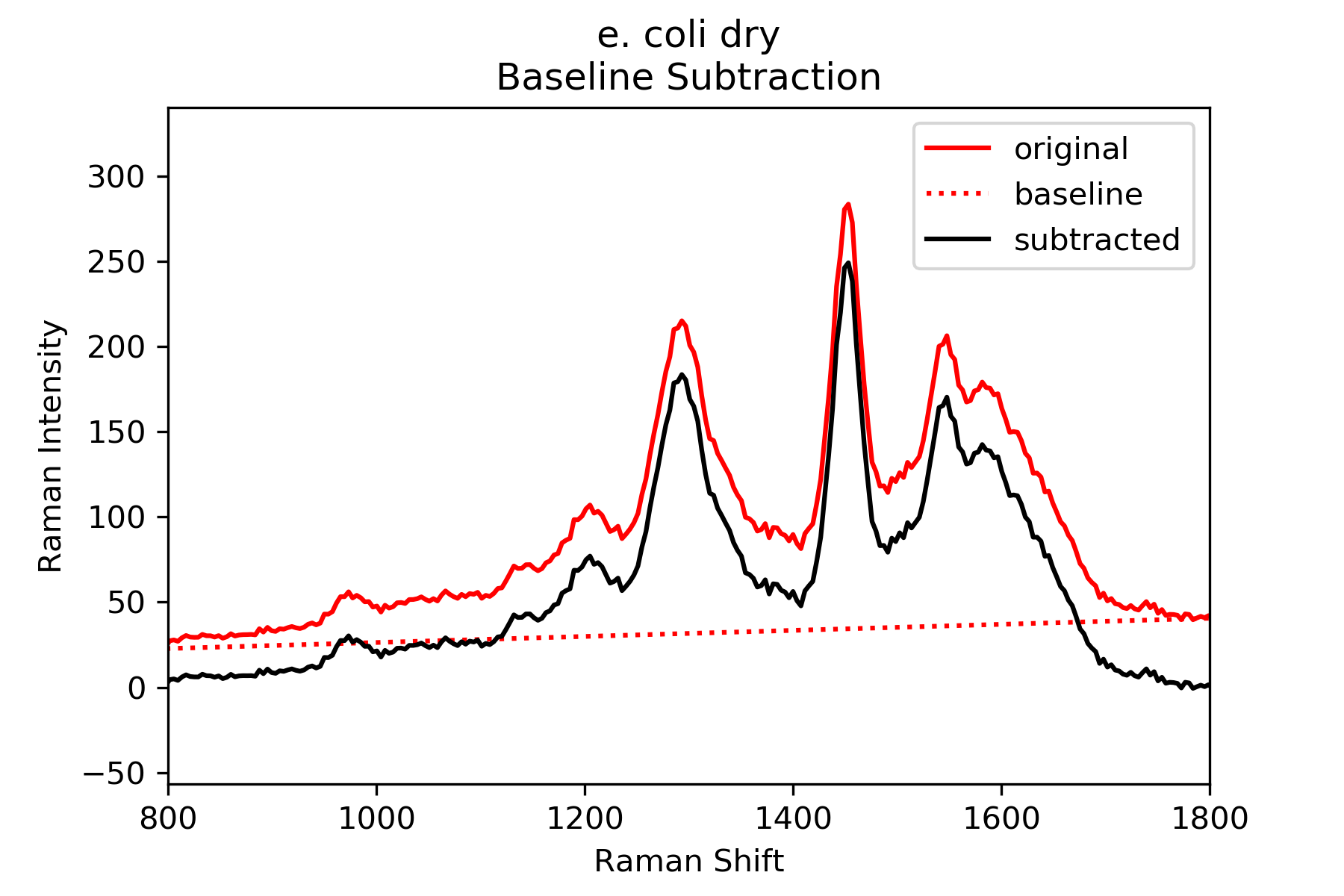

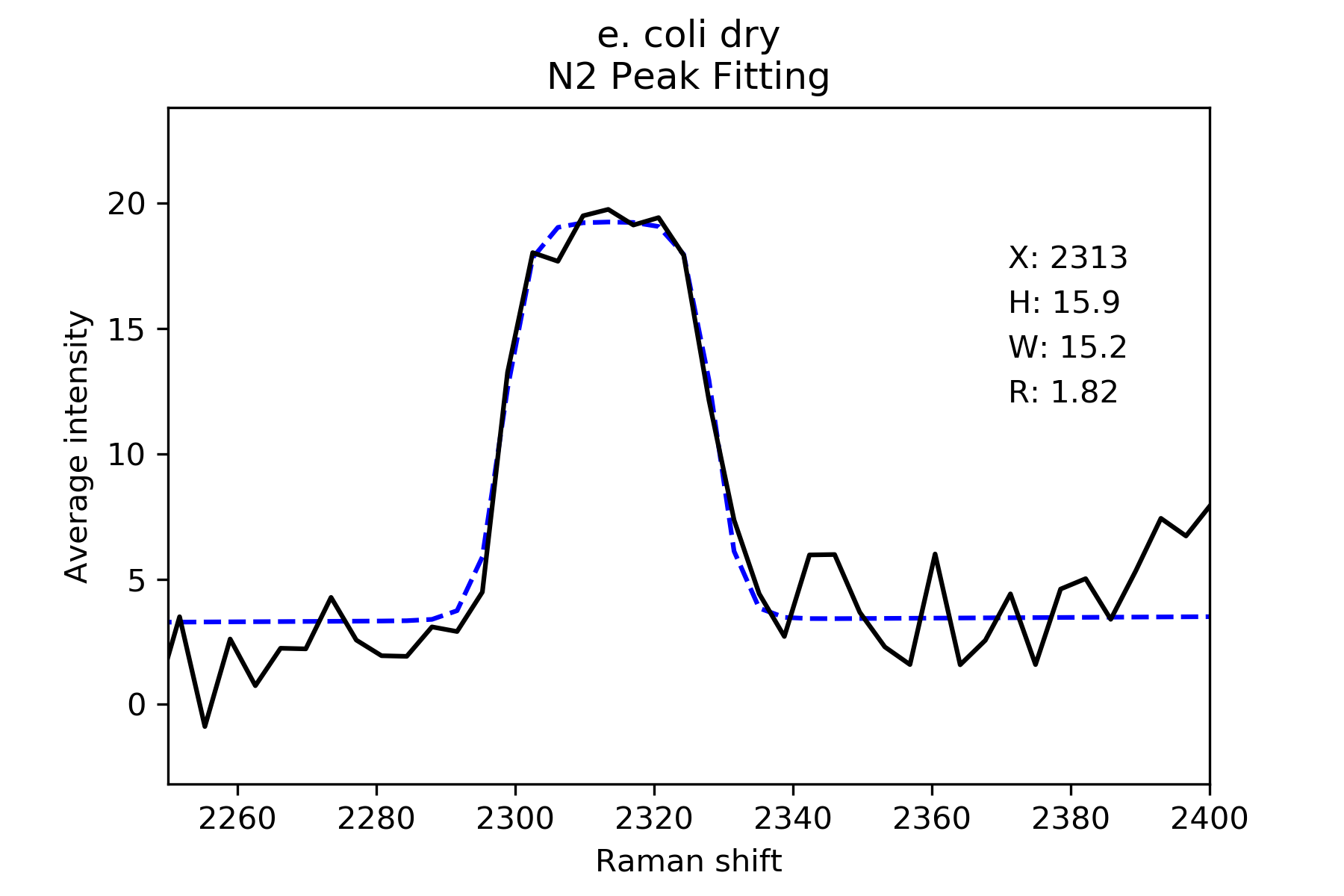

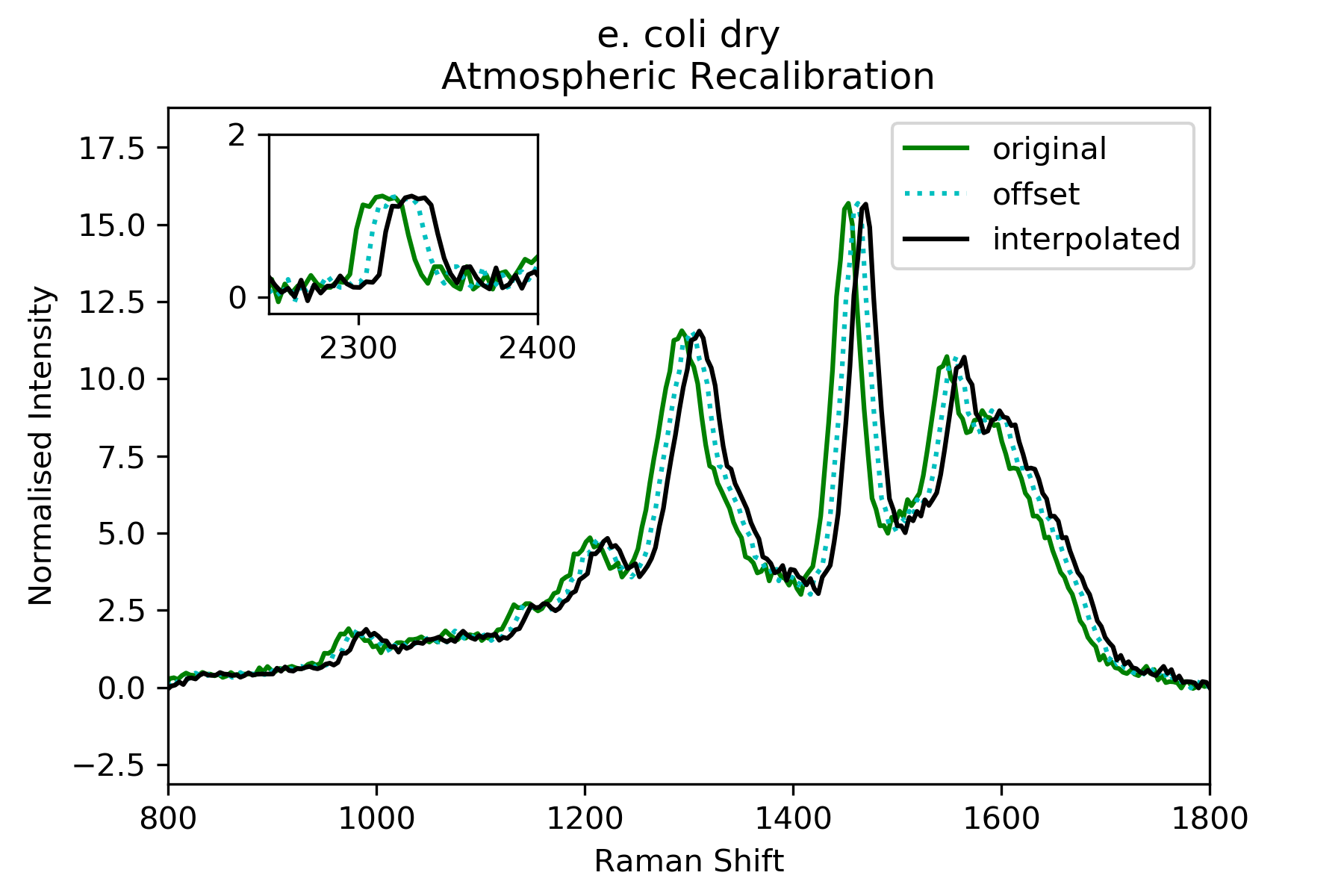

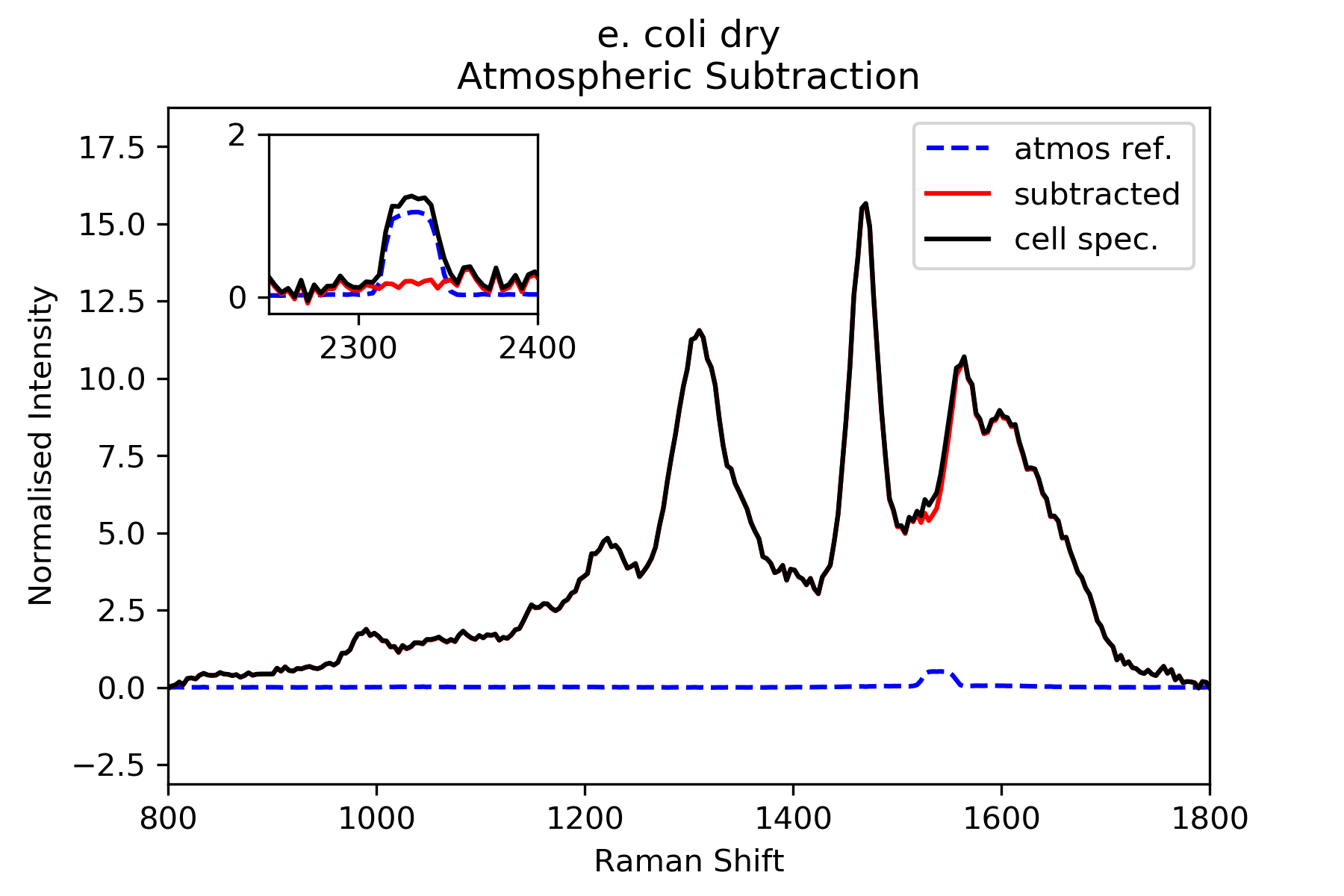


Figure S2: Post-processing of the DUV Raman spectrum of *E. coli*: linear baseline subtraction; N_2_ peak fitting; recalibration to a standard N_2_ peak position of 2331 cm^-1^; atmospheric peaks subtraction.


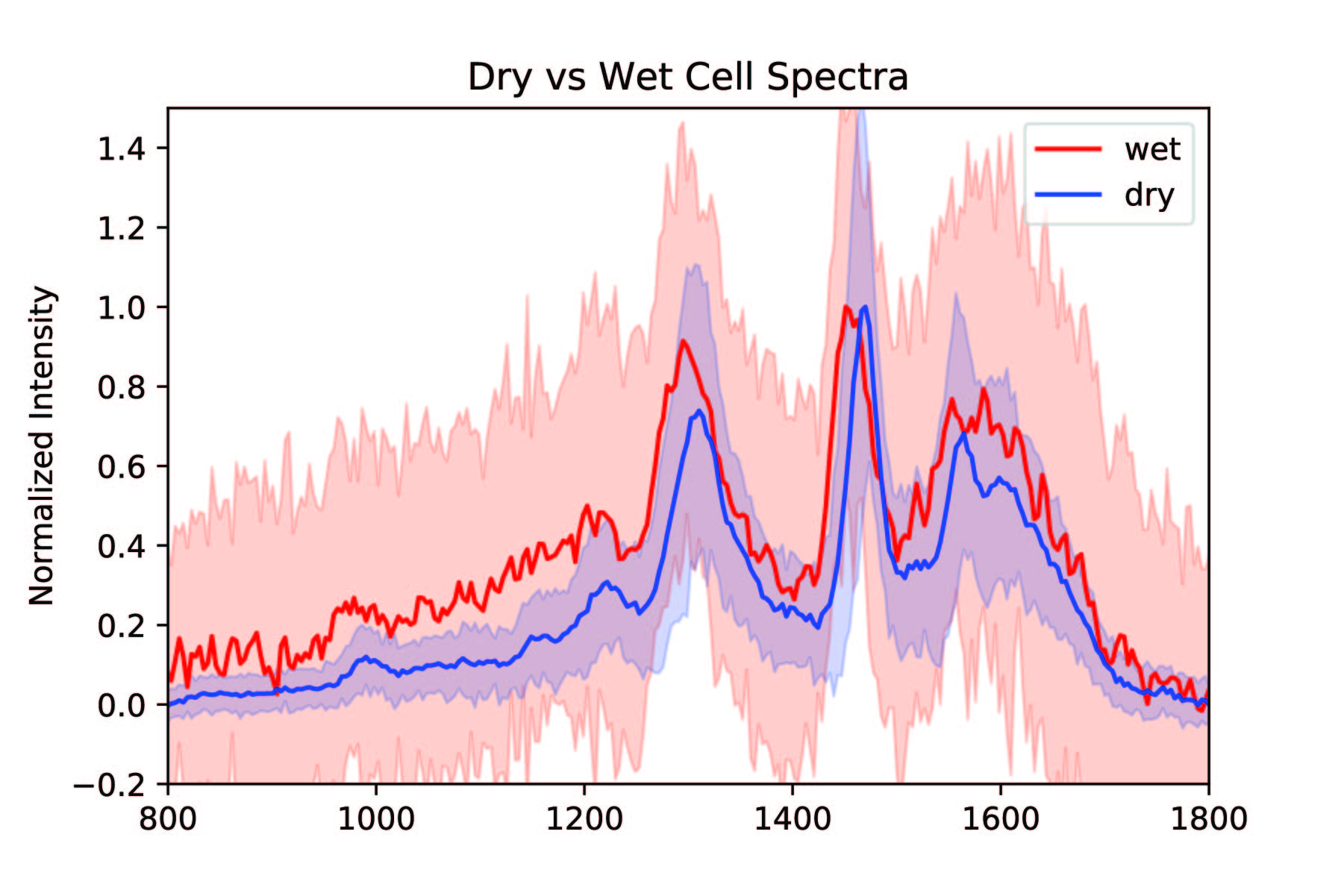


Figure S3: DUV Raman spectra for cells of *E. coli* during log phase, in solution and after being dried by spotting onto a sterile Al wafer. Shaded area indicates µ ± 2s.d..


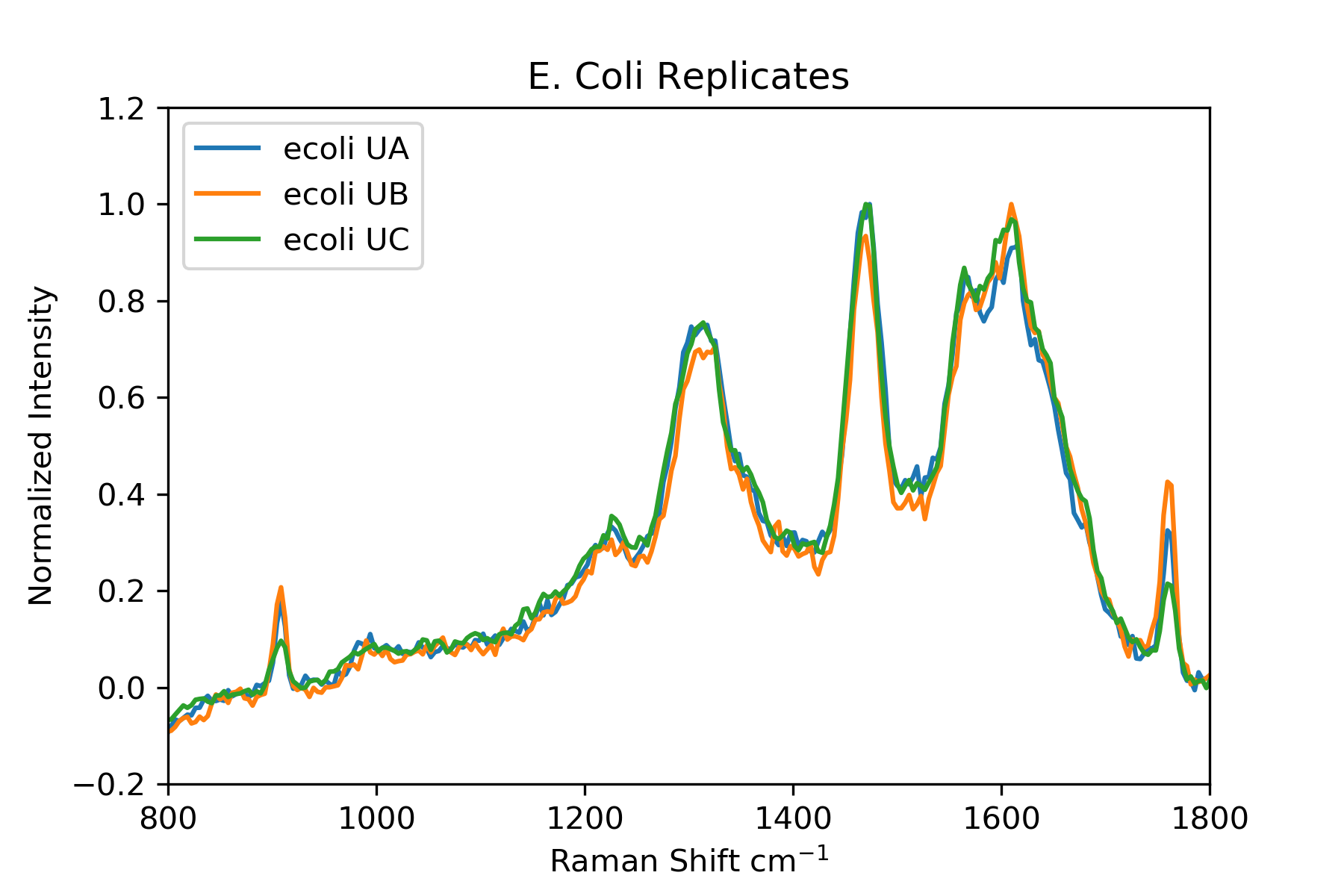


Figure S4: DUV Raman spectra for replicates of *E. coli*. Sharp peaks at ~910 and 1770 cm^-1^ are secondary laser lines reflected by the substrate.


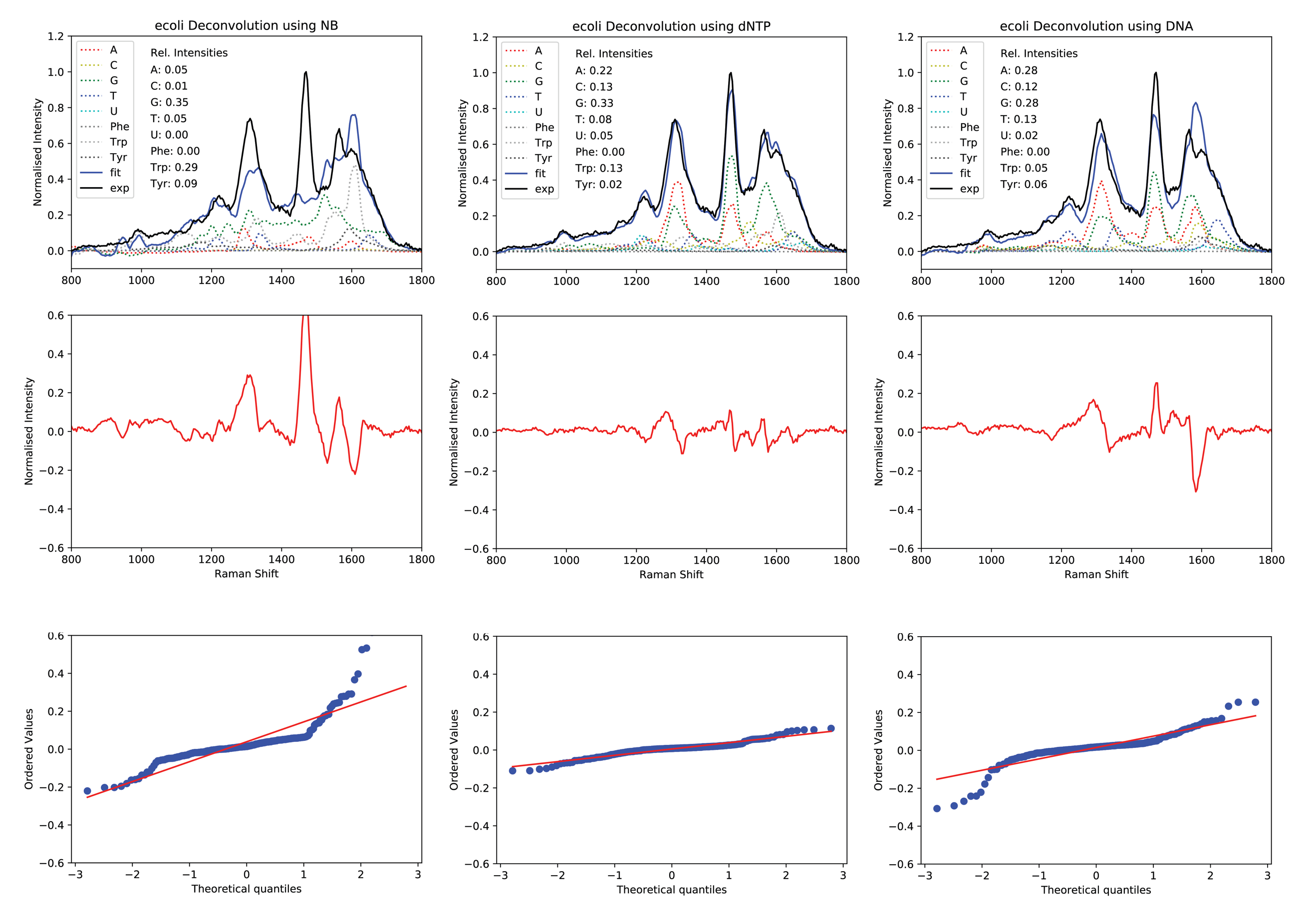


Figure S5: Residuals from deconvolution of the DUV Raman spectrum of *E. coli*, using nucleobase and amino acid spectra; using nucleotide and amino acid spectra; using ssDNA and amino acid spectra.


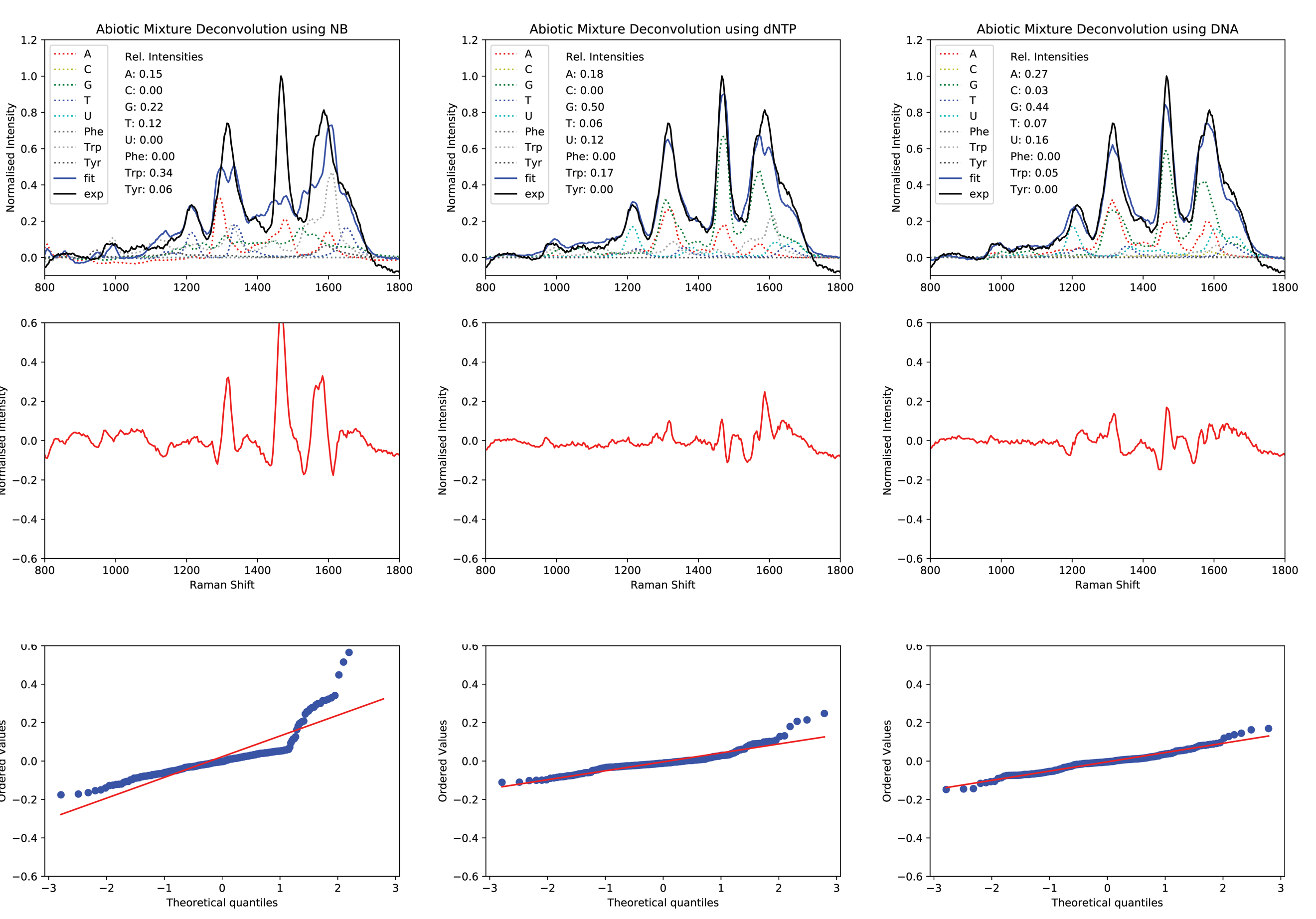


Artificial Mixture Deconvolution using DNA

Artificial Mixture Deconvolution using dNTP

Artificial Mixture Deconvolution using NB

Figure S6: Residuals from deconvolution of the DUV Raman spectra of the artificial mixture, using nucleobase and amino acid spectra; using nucleotide and amino acid spectra; using ssDNA and amino acid spectra.


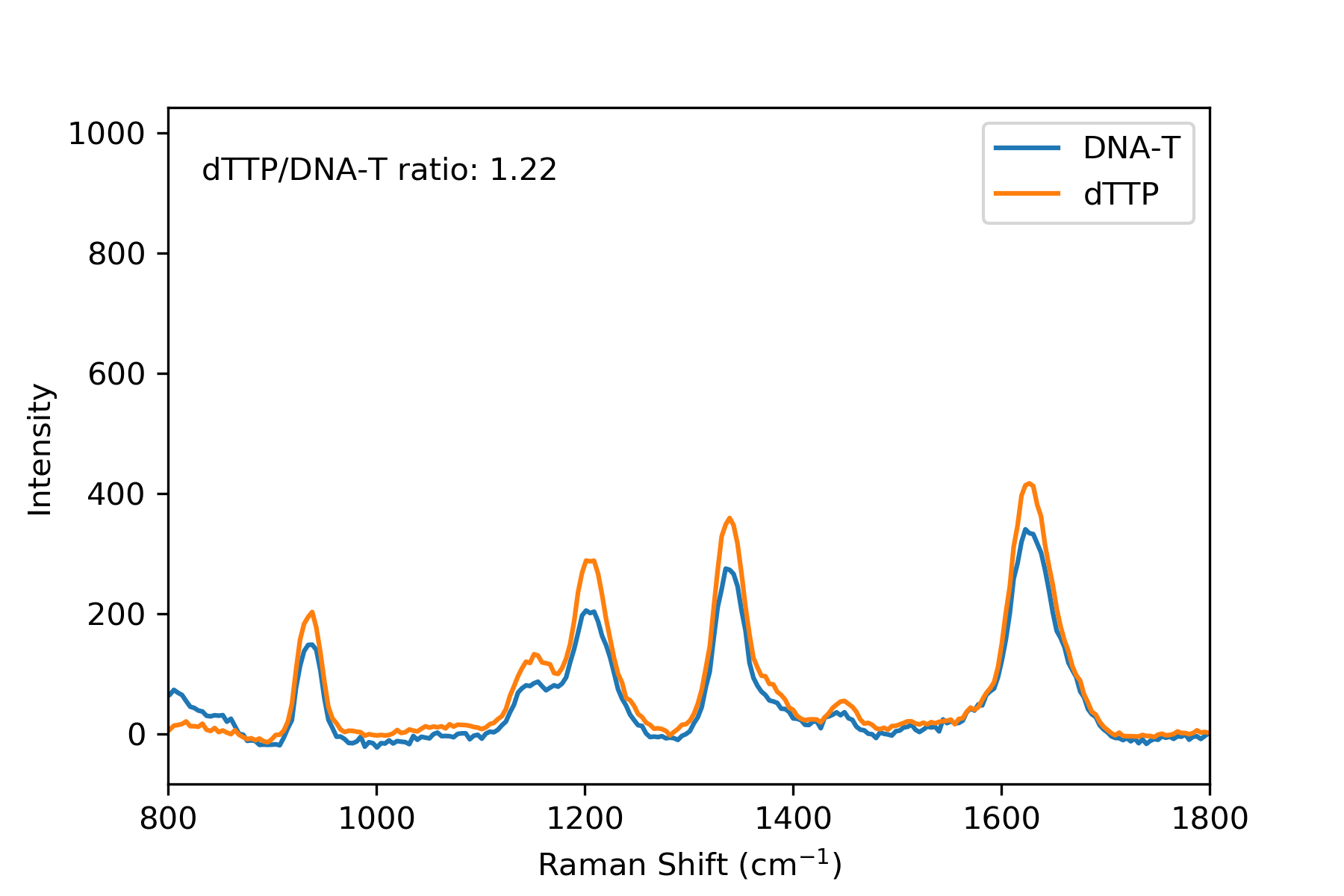


Figure S7: DUV Raman spectra of DNA-T and dTTP solutions with matching nucleotide concentrations of 0.5 mM, under 248 nm excitation and normalized to laser intensity, in order to compare the absolute intensities obtained from free nucleotide and nucleic acid at the same concentration.

| Component | Peak Position (cm^-1^) | | | | | | | |
| --- | --- | --- | --- | --- | --- | --- | --- | --- |
| Adenine^a^ |  | **C8H (b) C8N7 (s)** | C1N9 (s) C6N7 (s) | C2H (s) N9C8 (s) | C4N3 (s) C5C4 (s) |  |  |  |
| A |  | **1291** |  | 1477 | 1594 |  |  |  |
| dATP | 1233 | **1322** | 1413 | 1474 | 1576 |  |  |  |
| DNA-A | 1222 | **1314** | 1398 | 1470 | 1583 |  |  |  |
| Cytosine^a^ |  |  |  |  |  | **N3C4 (s)** |  | C4=O (s) C5C6 (s) |
| C | 1087 | 1207 | 1268 | 1341 | 1421 | **1512** |  | 1628 |
| dCTP | 1126 | 1237 |  | 1360 | 1405 | **1523** |  | 1643 |
| DNA-C | 1118 | 1164 | 1241 | 1394 |  | **1508** | 1587 |  |
| Guanine^a^ |  |  |  |  | **C8H (s) N9C8 (s)** |  | N3C4 (s) |  |
| G | 1199 | 1245 | 1310 |  | **1440** | 1523 | 1561 | 1685 |
| dGTP |  |  | 1306 | 1390 | **1474** |  | 1572 |  |
| DNA-G |  |  | 1314 |  | **1462** |  | 1572 |  |
| Thymine^a^ |  |  | C2N3 (s) | C1N9 (s) C6N7 (s) | **C4=O (s) C5C7 (s)** |  |  |  |
| T | 1141 | 1214 | 1337 |  | **1647** |  |  |  |
| dTTP | 1172 | 1226 | 1360 | 1462 | **1651** |  |  |  |
| DNA-T | 1155 | 1209 | 1343 | 1449 | **1634** |  |  |  |
| Uracil^b^ | **C5H (b) N3C4 (s)** | N3H (b) C6H (b) |  | C5C6 (s) C6H (b) | C4=O (s) |  |  |  |
| U | **1210** | 1367 |  |  | 1666 |  |  |  |
| UTP | **1214** | 1383 | 1459 | 1617 | 1674 |  |  |  |
| RNA-U | **1203** | 1367 | 1451 | 1602 | 1659 |  |  |  |
| Phenylalanine^c^ | Symm. Ring (s) | Phenyl-C (s) |  |  |  |  | **In-plane Ring (s)** |  |
| Phe | 940 | 1153 | 1260 |  |  |  | **1595** |  |
| Tryptophan^c^ | Symm. Purine (s) | Symm. Purine (s) |  | Pyrrole Ring (s) |  | Symm. Ring (s) | **Benzene (s)** |  |
| Trp | 990 | 1122 | 1226 | 1329 | 1447 | 1553 | **1610** |  |
| Tyrosine^c^ |  | CH (b) Phenyl-C (s) |  |  |  |  | **In-plane Ring (s)** |  |
| Tyr | 947 | 1176 |  |  |  |  | **1591** |  |

Table S1: Peak positions (in cm^-1^) for each molecular standard, arranged according to their assignments to known vibrational modes (s: stretching, b: bending) based on the literature: a) Wen *et al.*, 1997, b) Sun *et al.*, 2014, c) Asher *et al.*, 1986. Dominant modes for each group are in bold.

| **Nucleobase + AAA** | **A** | **C** | **G** | **T** | **U** | **Phe** | **Trp** | **Tyr** |
| --- | --- | --- | --- | --- | --- | --- | --- | --- |
| A |  | -0.23 | -0.35 | 0.00 | -0.11 | 0.14 | 0.07 | 0.27 |
| C |  |  | -0.30 | -0.36 | -0.13 | -0.10 | -0.25 | -0.15 |
| G |  |  |  | 0.01 | 0.31 | -0.03 | -0.24 | -0.01 |
| T |  |  |  |  | 0.68 | 0.01 | 0.06 | -0.01 |
| U |  |  |  |  |  | 0.21 | -0.04 | 0.18 |
| Phe |  |  |  |  |  |  | -0.22 | 0.86 |
| Trp |  |  |  |  |  |  |  | -0.40 |
| **Nucleotide + AAA** | **A** | **C** | **G** | **T** | **U** | **Phe** | **Trp** | **Tyr** |
| A |  | 0.12 | -0.61 | -0.25 | 0.08 | 0.22 | -0.11 | 0.23 |
| C |  |  | -0.33 | -0.39 | -0.05 | 0.10 | -0.27 | 0.05 |
| G |  |  |  | 0.13 | 0.03 | -0.22 | -0.23 | -0.20 |
| T |  |  |  |  | -0.68 | -0.12 | 0.16 | -0.17 |
| U |  |  |  |  |  | -0.19 | -0.11 | -0.14 |
| Phe |  |  |  |  |  |  | -0.20 | 0.88 |
| Trp |  |  |  |  |  |  |  | -0.36 |
| **DNA + AAA** | **A** | **C** | **G** | **T** | **U** | **Phe** | **Trp** | **Tyr** |
| A |  | -0.10 | -0.64 | -0.14 | 0.07 | -0.15 | -0.17 | 0.14 |
| C |  |  | -0.21 | -0.15 | -0.16 | 0.08 | -0.59 | -0.14 |
| G |  |  |  | 0.05 | -0.01 | 0.10 | 0.00 | -0.03 |
| T |  |  |  |  | -0.69 | -0.04 | 0.21 | 0.04 |
| U |  |  |  |  |  | 0.33 | -0.10 | -0.34 |
| Phe |  |  |  |  |  |  | 0.06 | -0.89 |
| Trp |  |  |  |  |  |  |  | -0.16 |

Table S2: Correlation coefficients from deconvolution of the DUV Raman spectrum of *E. coli*, using nucleobase and amino acid spectra; using nucleotide and amino acid spectra; using ssDNA and amino acid spectra.

| **Group** | **Molecular mass**  **(g/mol)** | **weight per cell (fg)** | **%mol in macromolecule** | **molecules per cell (x10^6^)** | **% dry weight** |
| --- | --- | --- | --- | --- | --- |
| **Protein ^1^** | **33,121** | **165** | **100** | **821** | **16.5** |
| Ala | 89.094 | 11.6 | 9.52 | 78.1 | 3.2 |
| Arg | 174.203 | 13.1 | 5.51 | 45.2 | 3.6 |
| Asn | 132.119 | 7.1 | 3.95 | 32.4 | 2.0 |
| Asp | 133.104 | 9.3 | 5.15 | 42.3 | 2.6 |
| Cys | 121.154 | 1.9 | 1.16 | 9.5 | 0.5 |
| Glu | 14.131 | 1.1 | 5.76 | 47.3 | 0.3 |
| Gln | 146.146 | 8.8 | 4.44 | 36.4 | 2.5 |
| Gly | 75.067 | 7.5 | 7.37 | 60.5 | 2.1 |
| His | 155.156 | 4.8 | 2.27 | 18.6 | 1.3 |
| Ile | 131.175 | 10.7 | 6.01 | 49.3 | 3.0 |
| Leu | 131.175 | 19.1 | 10.67 | 87.6 | 5.3 |
| Lys | 146.189 | 8.8 | 4.41 | 36.2 | 2.4 |
| Met | 149.208 | 5.7 | 2.82 | 23.1 | 1.6 |
| Phe | 165.192 | 8.8 | 3.89 | 31.9 | 2.4 |
| Pro | 115.132 | 7.0 | 4.43 | 36.4 | 1.9 |
| Ser | 105.093 | 8.3 | 5.8 | 47.6 | 2.3 |
| Thr | 119.119 | 8.8 | 5.4 | 44.3 | 2.4 |
| Trp | 204.228 | 4.3 | 1.53 | 12.6 | 1.2 |
| Tyr | 181.191 | 7.0 | 2.85 | 23.4 | 2.0 |
| Val | 117.148 | 11.3 | 7.07 | 58.0 | 3.2 |
| **RNA ^2^** |  | **60** | **100** | **106** | **6** |
| A | 347.22 | 15.1 | 24.8 | 26.2 | 4.2 |
| C | 323.2 | 12.4 | 21.8 | 23.0 | 3.5 |
| G | 363.22 | 20.6 | 32.4 | 34.2 | 5.8 |
| U | 324.18 | 11.9 | 21.0 | 22.2 | 3.3 |
| **DNA ^3^** |  | **9** | **100** | **16.6** | **0.9** |
| A | 330 | 2.2 | 24.5 | 4.07 | 0.6 |
| C | 307 | 2.1 | 24.6 | 4.08 | 0.6 |
| G | 347 | 2.5 | 26.2 | 4.35 | 0.7 |
| T | 322 | 2.2 | 24.7 | 4.10 | 0.6 |
| **Metabolites ^4^** |  | **70** | **conc. (mM)** | **36.4** | **7** |
| A | *variable* | 10.14 | 16.0 | 10.7 | 2.8 |
| C | *variable* | 1.60 | 2.75 | 1.84 | 0.4 |
| G | *variable* | 3.25 | 5.85 | 3.91 | 0.9 |
| T | *variable* | 2.63 | 4.98 | 3.33 | 0.7 |
| U | *variable* | 13.99 | 22.7 | 15.2 | 3.9 |
| Phe | 165.192 | 0.0033 | 0.018 | 0.012 | 0.0009 |
| Trp | 204.228 | 0.0027 | 0.012 | 0.0008 | 0.0008 |
| Tyr | 181.191 | 0.0058 | 0.029 | 0.019 | 0.002 |
| Non Raman-active | *variable* | 20.2 | 63.6 | 42.5 | 10.7 |
| **Other** |  | **57** |  |  | **5.7** |
| lipid | 800 | 27 |  | 20 | 9 |
| Lipopolysaccharide | 8000 | 9 |  | 1 | 3 |
| Peptidoglycan | (1000)_n_ | 9 |  | (1) | 3 |
| Glycogen | 1,000,000 | 9 |  | 0.004 | 3 |
| Inorganic Ions |  | 3 |  |  | 1 |
| **Total** | **100** | **1000** |  | **224** | **Total** |

Table S3: Detailed breakdown of the composition of an average cell of *E. coli* during exponential growth with a doubling time of 40 minutes, based on a cellular volume of 0.9 μm^3^ (Neidhardt *et al.*, 1990; Neirlich *et al.*, 1990; Weickert *et al.*, 1998; Blattner *et al.*, 1997; Bennett *et al.*, 2009)

|  | **Stock Solution** |  | **Abiotic Mixture** |  | **Cell Equivalent** |
| --- | --- | --- | --- | --- | --- |
| **Component** | **Concentration (mM)** | **Volume (μL)** | **Concentration (mM)** | **Relative Concentration** | **Relative Concentration** |
| **Free Nucleotides** |  | **68** | **0.135** |  |  |
| dATP | 100 | 19.9 | 0.040 | 100% | 100% |
| dCTP | 100 | 3.9 | 0.008 | 20% | 19% |
| dGTP | 100 | 7.3 | 0.015 | 37% | 37% |
| dTTP | 100 | 6.2 | 0.012 | 31% | 31% |
| UTP | 100 | 30.6 | 0.061 | 154% | 153% |
| **Nucleic Acids** |  | **281** | **0.563** |  |  |
| DNA-A | 0.1 | 69.7 | 0.139 | 350% | 350% |
| DNA-C | 0.1 | 62.5 | 0.125 | 314% | 313% |
| DNA-G | 0.1 | 88.9 | 0.178 | 447% | 446% |
| DNA-T | 0.1 | 9.4 | 0.019 | 47% | 47% |
| RNA-U | 0.1 | 51.1 | 0.102 | 257% | 256% |
| **Amino Acids** |  | **151** | **0.301** |  |  |
| Phe | 25 | 73.4 | 0.147 | 369% | 368% |
| Trp | 13.7 | 22.5 | 0.045 | 113% | 113% |
| Tyr | 2.65 | 54.6 | 0.109 | 274% | 274% |
| **Total** |  | **500** | **1.000** |  |  |

Table S4: Composition of the abiotic mixture, prepared by combination of the molecular standards according to the relative concentration of their equivalent residues in an average cell of *E. coli* as reported in Table 3.
